# The causal effects of genetically determined immune cells on gynecologic malignancies: a Mendelian randomization study

**DOI:** 10.3389/fonc.2024.1371309

**Published:** 2024-04-30

**Authors:** Yan Li, Jingting Liu, Qiandan Wang, Yawei Zhou, Chunhua Zhang, Jianying Pei

**Affiliations:** ^1^ Department of Biochemistry and Molecular Biology, Medical College of Northwest Minzu University, Lanzhou, China; ^2^ Maternal and Child Health Care Research Center, Gansu Provincial Maternity and Child Care Hospital, Lanzhou, China

**Keywords:** gynecologic malignancies, immunophenotype, Mendelian randomization, causality, immune system

## Abstract

**Background:**

Evidence from observational studies suggested a connection between immune cells and gynecologic malignancies. To investigate potential causative associations between immunophenotype traits and gynecologic malignancies, we used a two-sample Mendelian randomization analysis.

**Methods:**

The genetic instrumental variables of 731 immunophenotypes of peripheral blood were obtained by the GWAS database; the GWAS data of common gynecologic cancers were obtained from FinnGen study. The main statistic method was the inverse-variance weighted method. We also used the weighted mode, weighted median, and MR Egger for evaluations. The MR Steiger directionality test was further used to ascertain the reverse causal relationship between immune cells and gynecologic cancers.

**Results:**

We identified 50 highly probable immunophenotypes and 65 possible ones associated with gynecologic malignancies. The majority of the B cell panel was protective factors in cervical cancer. However, there was a correlation found in the B cells panel with a probable factor associated with an elevated risk of endometrial cancer. Immunophenotypes in the monocyte panel were linked to a lower probability of ovarian cancer and vulvar cancer. All of the gynecologic cancers in our study had no statistically significant impact on immune cells, according to reverse MR analysis.

**Conclusion:**

Our study firstly emphasized the genetically predicted causality between immune cells and gynecologic malignancies. This knowledge will be critical to formulating the measures to prevent malignancies in female at risk in future clinical practice.

## 1 Introduction

Gynecological malignancies (including cervical cancer, endometrial cancer, ovarian cancer and vulvar cancer) are estimated with 1,353,361 new cases, account for 7% of total new case, and 654,448 cancer deaths (6.6%) globally by the 2020 GLOBOCAN Statistic (1). Of them, ovarian cancer is one of the deadliest gynecological malignancies that affect women (2), and cervical cancer makes up the greatest fraction with an incidence rate of 3.1%. Furthermore, the burden could be made worse by the world’s population expansion and the increasing prevalence of risk factors (3). It highlighted how urgently effective preventative measures must be developed in order reduce the burden of gynecological cancer on the general public’s health. As a consequence, a lot of work needs to be done to cultivate novel interventions in order to find new cases and raise patient survival rates.

Tumor metabolism involves multiple metabolic pathways, and cancer is a complex pathological disease with an abnormal metabolic profile. Cancer cells have a different metabolic profile due to changes in these signal transduction pathways and the enzymatic machinery that goes along with them (4, 5). Surgery, radiation, and chemotherapy are the primary methods used for the treatment in cancer. Recently, however, targeted treatment and immunotherapy have all become significant tools in the battle anti-cancer. Recent research has gradually demonstrated that immune cells in the tumor microenvironment (TME) predict overall survival and play a substantial part in the progression of gynecologic malignancies (6, 7). Since cervical cancer is brought on by a chronic human papillomavirus (HPV) infection, it has been referred to as an immunogenic tumor. Myeloid-derived suppressor cells (MDSCs) created a premetastatic microenvironment in cervical cancer by expressing high levels of Cxcl2, S100a8/9, Bv8, and MMP-9. This niche promotes visceral organ metastasis (8). Tumor-infiltrating MDSCs and arginase-1 expression were also elevated in endometrial cancer (9). According to a study, activated memory CD4^+^ T cells and mast cells were independent predictors of overall survival for patients with cervical cancer. The majority of tumor-infiltrating immune cells (TICs) in cervical cancer were found to be CD8^+^ T cells and macrophages (10). According to a retrospective study, higher expression of Treg, M2 macrophages, and CD4 naïve T cells during immunotherapy was found to be predictive of worse overall survivals by Ni Y and colleagues. They also discussed the role of M2 macrophages, T-regulatory cells, and eosinophils, which are known to produce TGF-β in the TME (11). In a recent study, patients with cervical cancer who had higher levels of infiltration of naïve CD4^+^ T cells had a worse prognosis, but higher levels of M0 macrophage infiltration was associated with tumor stage and a better prognosis. The correlation between M0 macrophages and naïve CD4^+^ T cells was also confirmed by the results (12). Higher CD4^+^ (p = 0.0028) and CD45^+^ (p = 0.0221) infiltration was associated with a longer overall survival for patients with ovarian cancer, based on an observational study (13). However, most of the results mentioned above were obtained from observational or retrospective research, which might be constrained by the small sample size and heterogeneous patient organization. They had merely noted the connections between various immune cells and gynecologic cancers; it is unclear whether these relationships are causative. Owing to confounding factors and limited sample size, the results may be biased. Furthermore, the existing studies did not comprehensively investigate the associations between gynecologic malignancies and immunophenotype traits.

Mendelian randomization (MR), a statistical technique that has gained broad popularity, assesses the causal relationship between exposure and outcome by using genetic variants as instrumental variables (IVs) (14, 15). The MR analysis might not be affected by reverse causality and confounders because genetic variants are randomly distributed at conception (16).

Many MR studies that have been undertaken recently with a focus on gynecologic malignancies have emphasized the relationship between the risk of gynecologic cancers and lifestyle habits (e.g. coffee consumption (17), smoking (18, 19), alcohol consumption (18, 19), obesity (20), vitamin D (21)). Inspired by MR analysis’s non-confounding character, this study conducted the first thorough two-sample Mendelian randomization analysis to evaluate the causal relationships between immunophenotype traits and gynecologic cancers. Our findings may influence clinical practice, offer relevant risk factors and preventative hints. Our study aims to assist clinicians in identifying people who are very susceptible to gynecologic cancers, enabling more frequent follow-up and timely intervention.

## 2 Materials and methods

### 2.1 Study design

**Figure 1:**
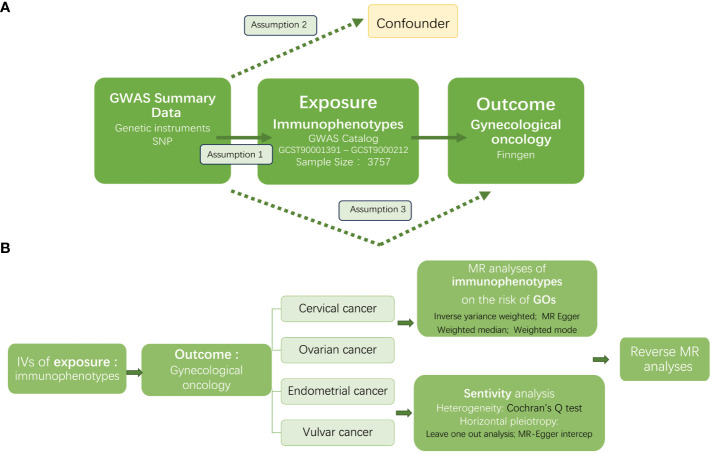
The workflow of this study design. GWAS, the genome wide association studies; SNPs, single-nucleotide polymorphisms; IVs, instrumental variables; GO, gynecologic oncology; MR, Mendelian randomization.

**Figure 1** displayed the overview of the study design. In this study we performed a Mendelian randomization (MR) analysis to assess the causal relationship between 731 immunophenotypes and gynecologic malignancies. Three important assumptions must be met when choosing instrumental variables (IVs) (**Figure 1A**): Three requirements must be met for the genetic variations to be considered as IVs: (1) they must be strongly correlated with the exposure; (2) they cannot be linked to any confounders; and (3) the variants chosen should only influence the risk of the outcome by the risk factor independently not through other pathways (22).

### 2.2 Exposure and outcome data sources

The 731 immunophenotypes of peripheral blood were published by the genome wide association studies (GWAS) and are accessible to the general public through the GWAS database(GCST90001391-GCST90002121) (23). The original GWAS on immunophenotypes used information from 3,757 European individuals. It contains 4 trait types,7 panels, and 731 traits. **Supplementary Table 1** provided an immunophenotype characterization. We selected four most common gynecologic malignancies including cervical cancer, ovarian cancer, endometrial cancer, and vulvar cancer as outcome. To obtain a more comprehensive conclusion of the causal links, we also took the carcinoma *in situ* into consideration. The GWAS summary data of the gynecologic cancer were accessed from the FinnGen study (24) (https://www.finngen.fi/en). **Table 1** presented the detailed information of datasets used in this study. The analysis was based on summary-level data from large genome wide association studies that were made available in public. Therefore, ethical approval was not needed.

**Table 1:** The GWAS datasets used for analyses.

Trait/Disease		Data Type	Consortium	Sample Size	Case	Control	GWAS_ID	Population
Immunophenotypes		Exposure	GWAS Catalog	3757	-	-	GCST90001391 - GCST9000212	European
Cervical cancer	Adenocarcinomas of cervix	Outcome	Finngen	167301	112	167189	finngen_R9_C3_CERVIX_ADENO_EXALLC	European
	Squamous cell neoplasms and carcinoma of cervix	Outcome	Finngen	167353	164	167189	finngen_R9_C3_CERVIX_SQUAM_EXALLC	European
	Malignant neoplasm of cervix uteri	Outcome	Finngen	167558	369	167189	finngen_R9_C3_CERVIX_UTERI_EXALLC	European
Endometrial cancer	Malignant neoplasm of corpus uteri	Outcome	Finngen	169156	1967	167189	finngen_R9_C3_CORPUS_UTERI_EXALLC	European
	Endometroid carcinoma of ovary	Outcome	Finngen	167411	222	167189	finngen_R9_C3_OVARY_ENDOMETROID_EXALLC	European
Ovarian cancer	Malignant neoplasm of ovary	Outcome	Finngen	168214	1025	167189	finngen_R9_C3_OVARY_EXALLC	European
	Serous carcinoma of ovary	Outcome	Finngen	168041	852	167189	finngen_R9_C3_OVARY_SEROUS_EXALLC	European
Vulvar cancer	Malignant neoplasm of vulva	Outcome	Finngen	167379	190	167189	finngen_R9_C3_VULVA_EXALLC	European
Carcinoma in situ	Carcinoma in situ of cervix uteri	Outcome	Finngen	167637	2236	165401	finngen_R9_CD2_INSITU_CERVIX_UTERI_EXALLC	European
	Carcinoma in situ of endometrium	Outcome	Finngen	167267	106	167161	finngen_R9_CD2_INSITU_ENDOMETRIUM_EXALLC	European
	Carcinoma in situ of vulva	Outcome	Finngen	167252	155	167097	finngen_R9_CD2_INSITU_VULVA_EXALLC	European

### 2.3 Instrumental variable selection

The IVs that met the strict significance threshold (P < 1 × 10^−8^) were chosen. SNPs with linkage disequilibrium were excluded concurrently (r^2^ > 0.001, window size < 10,000 kb). In order to minimize the bias caused by weak IVs, SNPs with F-statistics < 10 were also eliminated. Next, we looked through and eliminated SNPs corresponding to confounders via the PhenoScanner website (25). The confounders included (age at menarche (26), trunk fat mass, body mass index (27), obesity, treatment with ovestin 0.1% vaginal cream, and treatment with estrogen product). Finally, 251independent SNPs were obtained as IVs for immunophenotypes.

### 2.4 Statistical analysis

In measuring the causal relationships between immunophenotypes and gynecologic malignancies, four MR methods (inverse variance weighted (IVW) (28), MR Egger (29), Weighted median (30), and Weighted mode) were utilized, including. The primary analysis was the IVW. The Benjamini-Hochberg, which regulates the false discovery rate (FDR), was used to modify multiple testing. The heterogeneity was evaluated using the Cochran’s Q test. MR-Egger intercept and leave one out analysis were used to determine horizontal pleiotropy. Immunophenotypes with adj.P value <0.05 were deemed to have a highly probable relationship with gynecologic malignancies(statistically significant), while those that displayed P value <0.05 after MR analyzes, but 0.05< adj.P value <0.2 were considered possible factors. After excluding IVs that exhibited pleiotropic effects, we performed the primary MR analysis once more. To investigate if exposure was directionally causal for the outcome, we applied the MR Steiger directionality test (31). All analyses were carried out in R software 4.3.1 utilizing the “Two Sample MR” and “Mendelian Randomization” packages.

## 3 Results

### 3.1 Overview

For an insight into the relationship between immunophenotypes and four gynecologic cancers, we applied a tow-sample MR analysis. This study identified 252 independent SNPs linked to immunophenotypes (**Supplementary Table 2**). By applying IVW methods, there were 50 highly probable immunophenotype traits (adj.P value <0.05, **Tables 2**, **3**, **Supplementary Table 5**) and 65 possible immunophenotypes (P value <0.05, 0.05< adj.P value <0.2, **Supplementary Tables 4**, **5**) linked with gynecologic malignancies. When the highly probable immunophenotypes were classified in 7 panels (B cell, cDC, maturation stages of T cell, monocyte, myeloid cell, TBNK, Treg), 19 traits belonged to B cell, 8 from TBNK, 6 from cDC, monocyte, and maturation stages of T cell respectively, 4 from myeloid cell, and 2 from Treg. Sensitivity studies were performed to guarantee the robustness of the causal associations because IVW approaches are prone to weak IVs bias.

**Table 2:** The highly probable effects of immunophenotypes on cervical cancer by IVW method.

Outcome	Exposure	SNP(n)	OR (95%CI)	P value	adj.P value
Malignant neoplasm of cervix uteri	Terminally Differentiated CD4-CD8- T cell Absolute Count	2	5.989 ( 2.51, 14.29 )	5.470E-05	0.002
	FSC-A on Natural Killer T	2	0.235 ( 0.095, 0.584 )	0.002	0.027
Squamous cell neoplasms and carcinoma of cervix	Effector Memory CD4+ T cell %CD4+ T cell	3	2.131 ( 1.114, 4.077 )	0.022	0.046
	Effector Memory CD4-CD8- T cell %T cell	3	0.378 ( 0.163, 0.879 )	0.024	0.046
	Terminally Differentiated CD4-CD8- T cell Absolute Count	2	8.017 ( 2.232, 28.798 )	0.001	0.021
	BAFF-R on CD24+ CD27+ B cell	8	0.745 ( 0.581, 0.953 )	0.019	0.045
	BAFF-R on IgD+ CD24+ B cell	6	0.745 ( 0.613, 0.905 )	0.003	0.021
	BAFF-R on IgD+ CD24- B cell	10	0.769 ( 0.641, 0.923 )	0.005	0.021
	BAFF-R on IgD+ CD38- B cell	9	0.755 ( 0.626, 0.909 )	0.003	0.021
	BAFF-R on IgD+ CD38- naive B cell	8	0.768 ( 0.64, 0.92 )	0.004	0.021
	BAFF-R on IgD+ CD38+ B cell	10	0.761 ( 0.629, 0.92 )	0.005	0.021
	BAFF-R on IgD+ CD38dim B cell	10	0.763 ( 0.636, 0.915 )	0.004	0.021
	BAFF-R on IgD- CD38- B cell	7	0.758 ( 0.594, 0.966 )	0.025	0.047
	BAFF-R on memory B cell	7	0.734 ( 0.595, 0.904 )	0.004	0.021
	BAFF-R on naive-mature B cell	10	0.762 ( 0.634, 0.916 )	0.004	0.021
	BAFF-R on unswitched memory B cell	7	0.733 ( 0.596, 0.902 )	0.003	0.021
	BAFF-R on switched memory B cell	9	0.749 ( 0.591, 0.95 )	0.017	0.045
	BAFF-R on IgD+ B cell	10	0.764 ( 0.636, 0.917 )	0.004	0.021
	BAFF-R on transitional B cell	8	0.749 ( 0.612, 0.916 )	0.005	0.021
	CD19 on CD24+ CD27+ B cell	2	0.577 ( 0.369, 0.902 )	0.016	0.045
	CD19 on memory B cell	2	0.538 ( 0.325, 0.89 )	0.016	0.045
	CD20 on IgD+ CD38+ B cell	5	1.887 ( 1.078, 3.306 )	0.026	0.047
	BAFF-R on B cell	10	0.768 ( 0.638, 0.923 )	0.005	0.021
	CD3 on HLA DR+ CD4+ T cell	2	1.843 ( 1.085, 3.131 )	0.024	0.046
	CD3 on activated CD4 regulatory T cell	4	1.465 ( 1.05, 2.043 )	0.024	0.046
	CD3 on activated & secreting CD4 regulatory T cell	4	1.457 ( 1.05, 2.023 )	0.024	0.046
	CD123 on plasmacytoid Dendritic Cell	2	2.48 ( 1.229, 5.003 )	0.011	0.039
	CD123 on CD62L+ plasmacytoid Dendritic Cell	2	2.5 ( 1.231, 5.077 )	0.011	0.039
	CD80 on plasmacytoid Dendritic Cell	2	2.62 ( 1.244, 5.515 )	0.011	0.039
	CD80 on CD62L+ plasmacytoid Dendritic Cell	2	2.641 ( 1.246, 5.596 )	0.011	0.039
	SSC-A on plasmacytoid Dendritic Cell	5	2.508 ( 1.243, 5.059 )	0.010	0.039
	HLA DR on B cell	8	0.674 ( 0.499, 0.909 )	0.010	0.039

**Table 3:** The highly probable effects of immunophenotypes on ovarian and vulvar cancer by IVW method.

Outcome	Exposure	SNP(n)	OR (95%CI)	P value	adj.P value
Malignant neoplasm of ovary	HLA DR++ monocyte %leukocyte	2	0.648 ( 0.5, 0.84 )	0.001	0.011
	Monocytic Myeloid-Derived Suppressor Cells Absolute Count	5	1.205 ( 1.067, 1.361 )	0.003	0.018
	CD45 on B cell	2	1.855 ( 1.146, 3.001 )	0.012	0.046
	HLA DR on CD14+ CD16- monocyte	5	0.823 ( 0.74, 0.916 )	3.381E-04	0.006
	HLA DR on CD14+ monocyte	5	0.819 ( 0.734, 0.913 )	3.365E-04	0.006
	HLA DR on monocyte	4	0.824 ( 0.73, 0.93 )	0.002	0.016
	HLA DR on myeloid Dendritic Cell	6	0.862 ( 0.77, 0.964 )	0.009	0.042
	HLA DR on CD33+ HLA DR+ CD14dim	3	0.769 ( 0.646, 0.915 )	0.003	0.019
	HLA DR on CD33- HLA DR+	2	0.855 ( 0.76, 0.962 )	0.009	0.042
	HLA DR++ monocyte %leukocyte	2	0.443 ( 0.248, 0.793 )	0.006	0.036
	Terminally Differentiated CD4+ T cell Absolute Count	2	3.135 ( 1.247, 7.883 )	0.015	0.043
	CD20 on IgD+ CD24+ B cell	2	0.326 ( 0.139, 0.765 )	0.010	0.037
	CD4 on HLA DR+ CD4+ T cell	2	0.239 ( 0.099, 0.579 )	0.002	0.029
	HLA DR on CD14+ CD16- monocyte	5	0.69 ( 0.543, 0.876 )	0.002	0.029
	HLA DR on CD14+ monocyte	5	0.682 ( 0.533, 0.873 )	0.002	0.029
	HLA DR on monocyte	4	0.706 ( 0.537, 0.928 )	0.013	0.039
	HLA DR on CD33+ HLA DR+ CD14-	2	0.642 ( 0.466, 0.884 )	0.007	0.036
	HLA DR on B cell	9	1.391 ( 1.115, 1.736 )	0.003	0.032
Carcinoma in situ of vulva	Terminally Differentiated CD4-CD8- T cell Absolute Count	2	8.095 ( 2.206, 29.699 )	0.002	0.039

### 3.2 Cervical cancer

**Figure 2:**
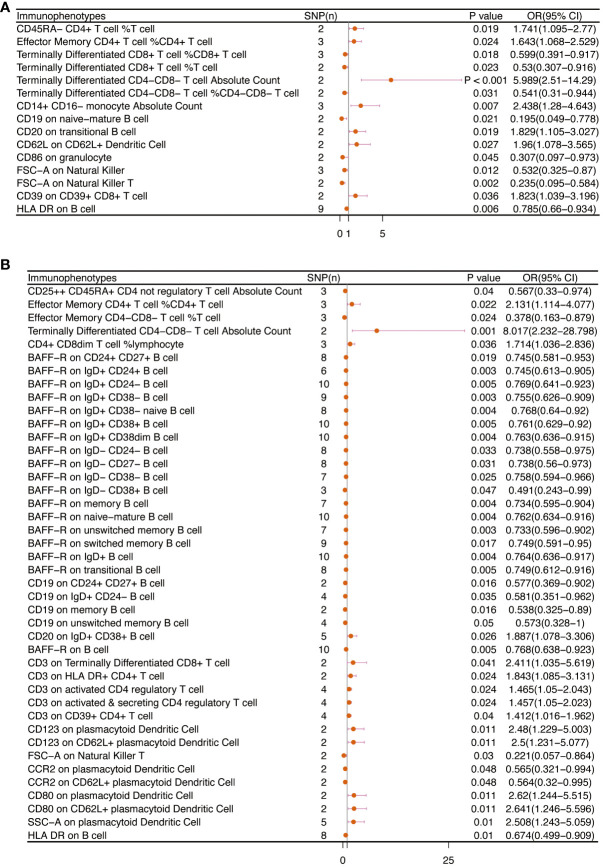
The causal effect of immunophenotypes on cervical cancer. **(A)** Adenocarcinomas of cervix; **(B)** Squamous cell neoplasms and carcinoma of cervix; SNP(n), the number of single-nucleotide polymorphisms; OR, odds ratio; CI, confidence interval.

**Figure 3:**
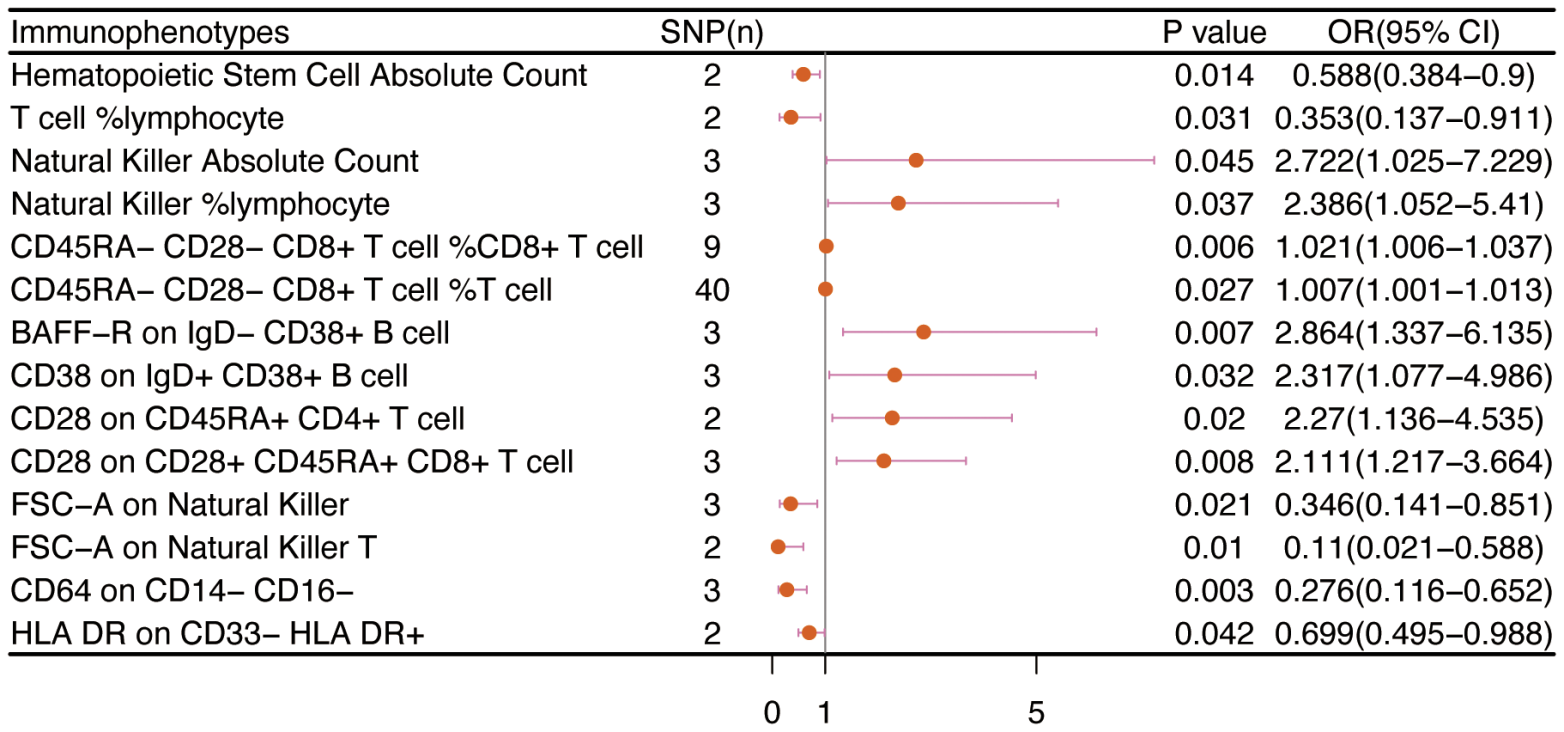
The causal effect of immunophenotypes on cervical cancer (Malignant neoplasm of cervix uteri). SNP(n), the number of single-nucleotide polymorphisms; OR, odds ratio; CI, confidence interval.

**Table 2** and **Figure 8** demonstrated that 32 pairs had highly probable causal effects (adj.P <0.05) between immunophenotypes and cervical cancer. The causal effect of immunophenotypes on adenocarcinomas of cervix was shown in **Figure 2A**. The B-cell panel showed the highest number of significant associations when compared to other panels, and the majority of B cells were protective factors against cervical cancer. In the study we conducted, B-cell activating factor receptor (BAFF-R) was the most frequently expressed molecule in our analysis. It expresses on several B cell subtypes, such as CD24^+^ CD27^+^ B cell, IgD^+^ CD24^+^ B cell, IgD^+^ CD24- B cell, IgD^+^ CD38^-^ B cell, memory B cell, and naive-mature B cell. However, CD20 on IgD+ CD38+ B cell (OR=1.887, 95%CI:1.078-3.306, P=0.026) had a significant association with the probability of cervical cancer (**Table 2**, **Figure 2B**). In cDC panels, CD123 on plasmacytoid Dendritic Cell (OR=2.48, 95%CI:1.229-5.003, P=0.011), CD123 on CD62L^+^ plasmacytoid Dendritic Cell (OR=2.5, 95%CI:1.231-5.077, P=0.011), CD80 on plasmacytoid Dendritic Cell (OR=2.62, 95%CI:1.244-5.515, P=0.011), CD80 on CD62L^+^ plasmacytoid Dendritic Cell (OR=2.641, 95%CI:1.246-5.596, P=0.011) had a disadvantageous association with cervical cancer(**Table 2**, **Figure 2B**). The causal effect of immunophenotypes on Malignant neoplasm of cervix uteri was shown in **Figure 3**.

### 3.3 Endometrial cancer

**Figure 4:**
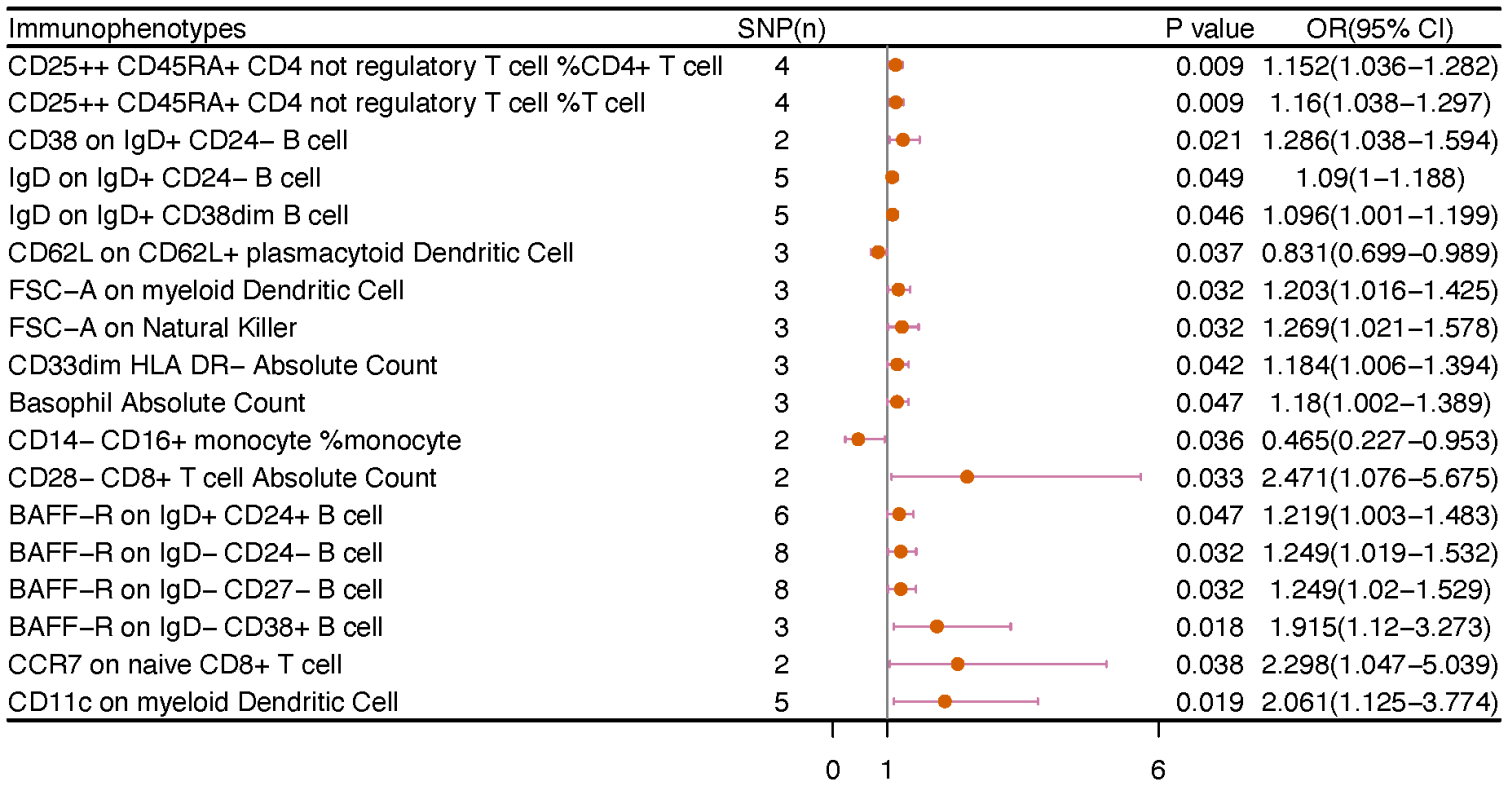
The causal effect of immunophenotypes on endometrial cancer. SNP(n), the number of single-nucleotide polymorphisms; OR, odds ratio; CI, confidence interval.

No immunophenotype was found at the criterion “adj.P value <0.05” after FDR correction. Nevertheless, 18 immunophenotypes that fit the requirements of P value <0.05 and 0.05< adj.P value <0.2 are possible factors in our study (**Figures 4**, **9**). B cells were also vital in endometrial cancer, similarly to how they were in cervical cancer. But all possible factors in B cell panel, namely, CD38 on IgD^+^ CD24^-^ B cell (OR=1.286, 95%CI; 1.038-1.594, P=0.021), IgD on IgD^+^ CD24- B cell (OR=1.09, 95%CI; 1-1.188, P=0.049), IgD on IgD^+^ CD38dim B cell (OR=1.096, 95%CI; 1.001-1.199, P=0.046), BAFF-R on IgD^+^ CD24^+^ B cell (OR=1.219, 95%CI; 1.003-1.483, P=0.047), BAFF-R on IgD- CD24- B cell (OR=1.249, 95%CI; 1.019-1.532, P=0.032), BAFF-R on IgD^-^ CD27^-^ B cell (OR=1.249, 95%CI; 1.02-1.529, P=0.032), BAFF-R on IgD- CD38^+^ B cell (OR=1.915, 95%CI; 1.12-3.273, P=0.018) were correlated with increased risk of endometrial cancer. In other panels, CD25^++^ CD45RA^+^CD4^+^ T cell, CD28^-^ CD8^+^ T cell, CCR7 on naive CD8^+^ T cell, myeloid Dendritic Cell, Natural Killer cell, Basophil cell had positive links with endometrial cancer. However, CD62L on CD62L^+^ plasmacytoid Dendritic Cell (OR=0.831, 95%CI; 0.699-0.989, P=0.037) and CD14^-^ CD16^+^ monocyte %monocyte (OR=0.465, 95%CI; 0.227-0.953, P=0.036) were negatively associated with the risk of endometrial cancer.

### 3.4 Ovarian cancer

**Figure 5:**
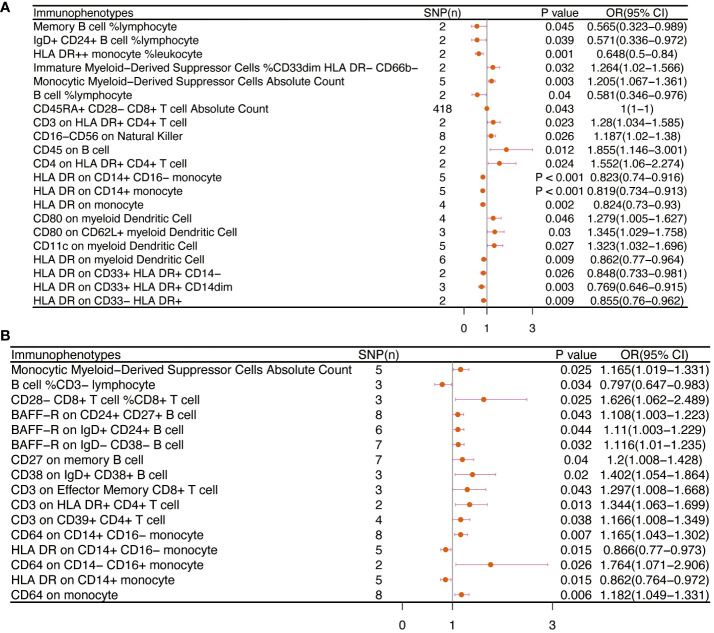
The causal effect of immunophenotypes on ovarian cancer. **(A)** Malignant neoplasm of ovary; **(B)** Serous carcinoma of ovary; SNP(n), the number of single-nucleotide polymorphisms; OR, odds ratio; CI, confidence interval.

We noticed 9 highly probable immunophenotype traits were connected to ovarian cancer in IVW test (**Figure 8**, **Table 3**). In the Monocyte panel, reduction in the risk of ovarian cancer was associated with HLA DR on CD14^+^ CD16- monocyte (OR=0.823, 95%CI:0.74-0.916, P=3.381E-04), HLA DR on CD14^+^ monocyte (OR=0.819, 95%CI:0.734-0.913, P=3.365E-04), and HLA DR on monocyte (OR=0.824, 95%CI:0.73-0.93, P=0.002). However, immune cells of different traits had opposing effects on ovarian cancer in TBNK and Myeloid cell panels. HLA DR^++^ monocyte, HLA DR on CD33^+^ HLA DR^+^ CD14dim, and HLA DR on CD33^-^ HLA DR^+^ exhibited favorable effects. On the other hand, Monocytic Myeloid-Derived Suppressor Cells and CD45 on B cell were positively associated with the increasing risk of ovarian cancer. The causal effect of immunophenotypes on ovarian cancer was shown in **Figure 5**.

### 3.5 Vulvar cancer

**Figure 6:**
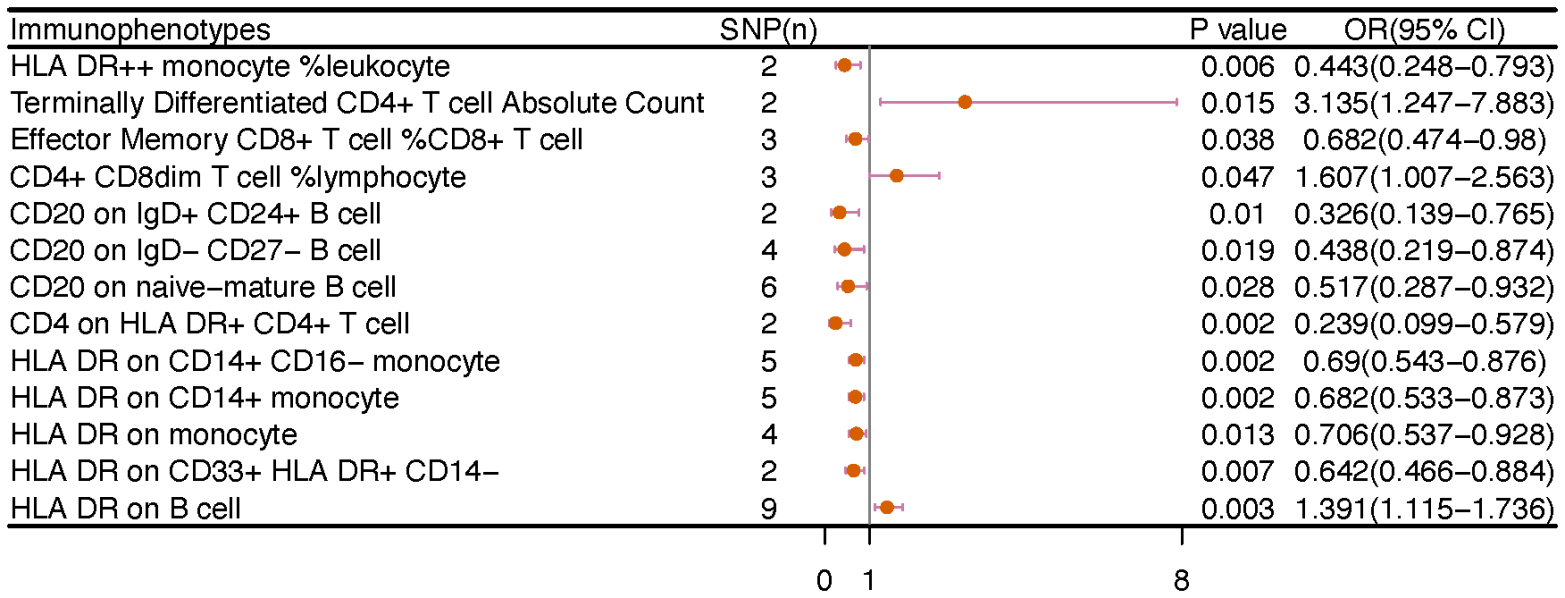
The causal effect of immunophenotypes on vulvar cancer. SNP(n), the number of single-nucleotide polymorphisms; OR, odds ratio; CI, confidence interval.

The IVW analysis indicated that 9 highly probable immunophenotype traits had causal relationship with vulvar cancer (**Figure 8**, **Table 3**). Terminally Differentiated CD4^+^ T cell Absolute Count (OR=3.135, 95%CI:1.247-7.883, P=0.015) and HLA DR on B cell (OR=1.391, 95%CI:1.115-1.736, P=0.003) were correlated with increased vulvar cancer risk. Whereas HLA DR^++^ monocyte %leukocyte (OR=0.443, 95%CI:0.248-0.793, P=0.006), CD20 on IgD^+^ CD24^+^ B cell (OR=0.326, 95%CI:0.139-0.765, P=0.010), CD4 on HLA DR^+^ CD4^+^ T cell (OR=0.239, 95%CI:0.099-0.579, P=0.002), HLA DR on CD14^+^ CD16^-^ monocyte (OR=0.69, 95%CI:0.543-0.876, P=0.002), HLA DR on CD14^+^ monocyte (OR=0.682, 95%CI:0.533-0.873, P=0.002), HLA DR on monocyte (OR=0.706, 95%CI:0.537-0.928, P=0.013), HLA DR on CD33^+^ HLA DR^+^ CD14^-^ (OR=0.642, 95%CI:0.466-0.884, P=0.007) were related to a reduced risk of vulvar cancer. In addition, we also screened 5 possible immunophenotypes for vulvar cancer. **Figure 6** and **Supplementary Table 3** presented the detailed data.

### 3.6 Gynecologic carcinoma *in situ*

There are 65 possible immunophenotypes for gynecologic cancer *in situ*, but we found only one highly probable immunophenotype trait after FDR correction. There are 65 possible immunophenotypes for gynecologic cancer in situ, but we found only one highly probable immunophenotype trait after FDR correction (**Figure 10**). Terminally Differentiated CD4^-^CD8^-^ T cell (OR=8.095, 95CI%: 2.206-29.699, adj.P= 0.039) was significantly positive link with carcinoma *in situ* of vulva risk.

**Figure 7:**
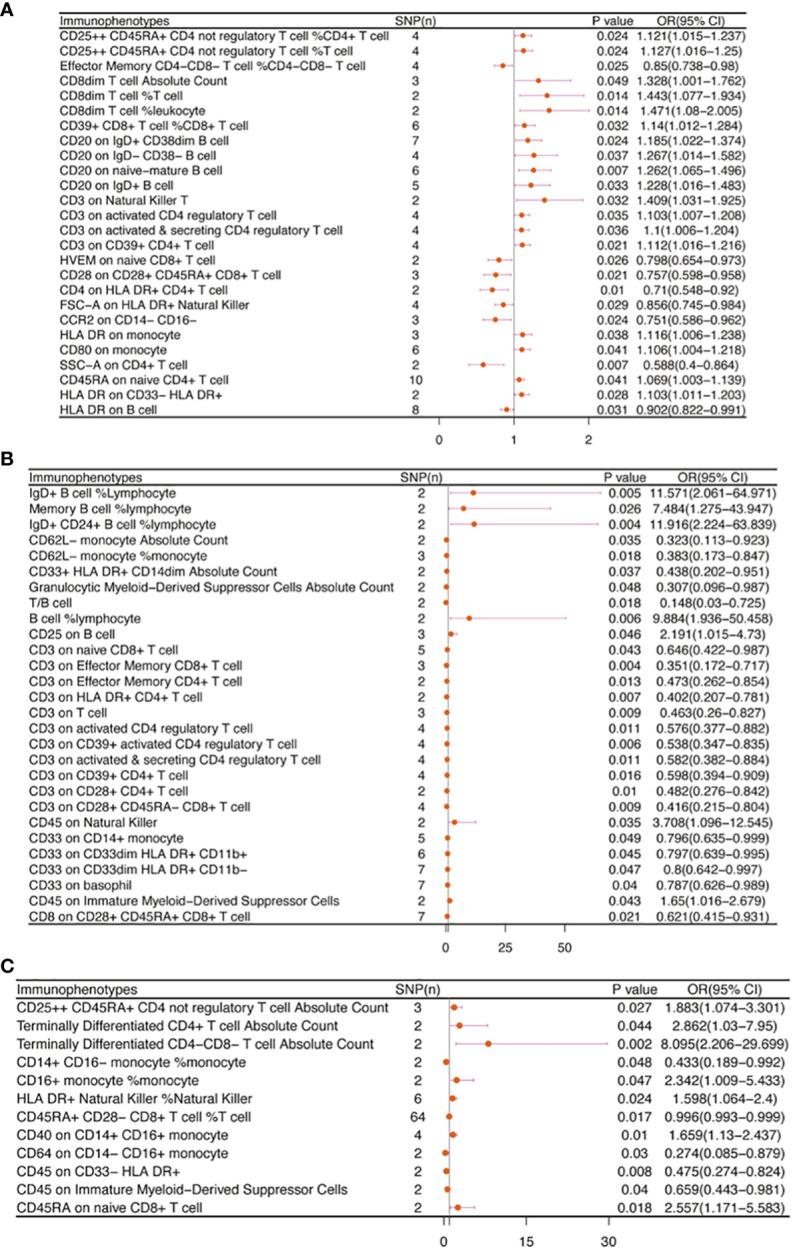
The causal effect of immunophenotypes on gynecologic carcinoma *in situ*. **(A)** Carcinoma *in situ* of cervix uteri; **(B)** Carcinoma *in situ* of endometrium; **(C)** Carcinoma *in situ* of vulva; SNP(n), the number of single-nucleotide polymorphisms; OR, odds ratio; CI, confidence interval.

In carcinoma *in situ* of cervix uteri, 26 causative links between the immune cells and cervical carcinoma *in situ* have been identified (**Figure 7A**, **Supplementary Table 5**). Compared to other panels, TBNK had the greatest number of possible associations. CD8dim T cell was linked in a higher incidence of cervical carcinoma *in situ*, but NKT cell, HLA DR^+^ CD4^+^ T cell, and HLA DR^+^ NK cell were protective immune factors. Treg cells also play an important role. CD39^+^ CD8^+^ T cell %CD8^+^ T cell (OR=1.14, 95%CI:1.012-1.284, P=0.032), CD3 on activated CD4 regulatory T cell (OR=1.103, 95%CI:1.007-1.208, P=0.035), CD3 on activated & secreting CD4 regulatory T cell (OR=1.1, 95%CI:1.006-1.204, P=0.036), CD3 on CD39^+^ CD4^+^ T cell (OR=1.112, 95%CI:1.016-1.216, P=0.021) were linked in a higher incidence of cervical carcinoma *in situ*.

**Figure 8:**
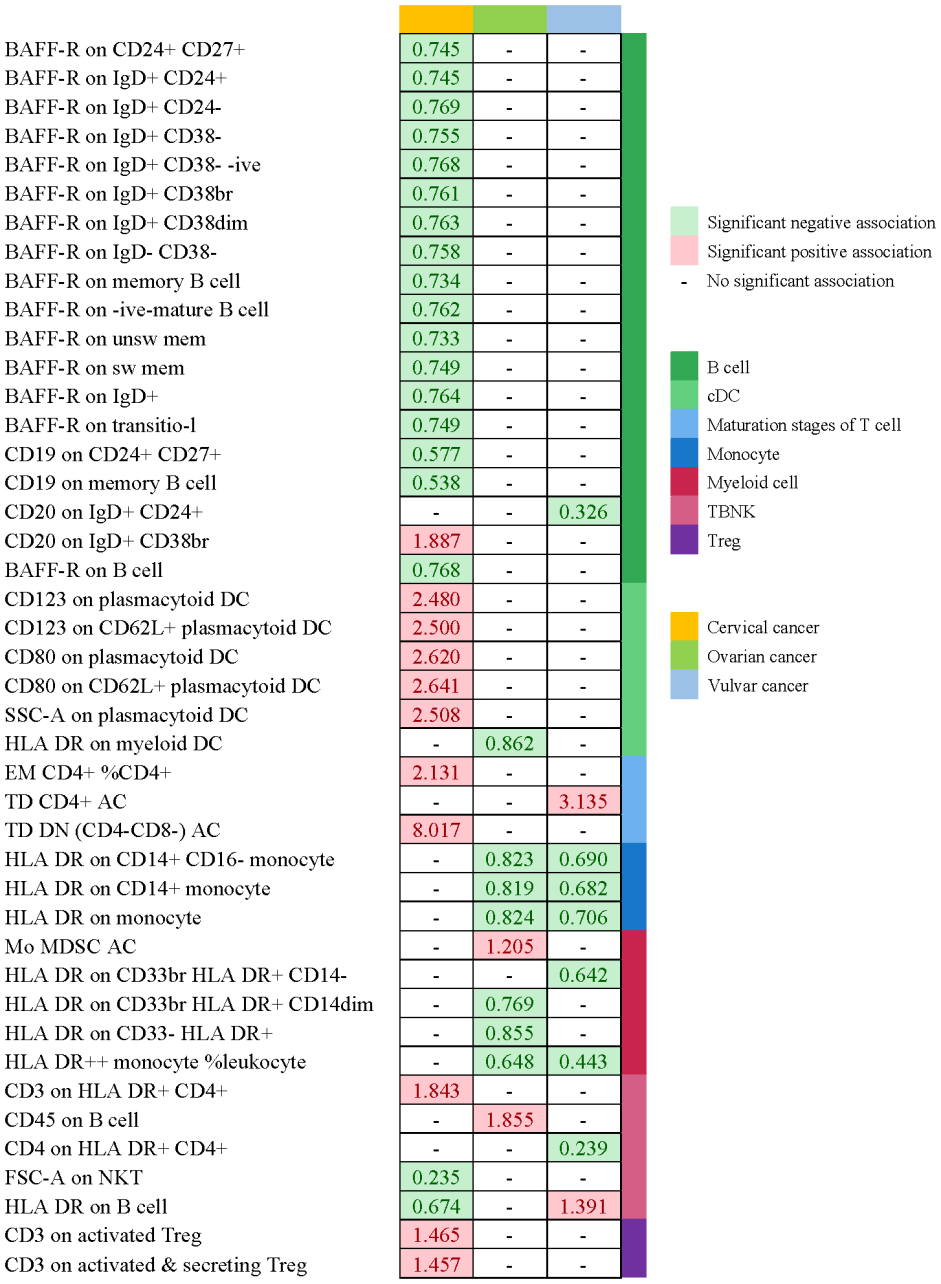
Summary of associations of genetically predicted immunophenotype traits with cervical cancer, ovarian cancer and vulvar cancer.

**Figure 9:**
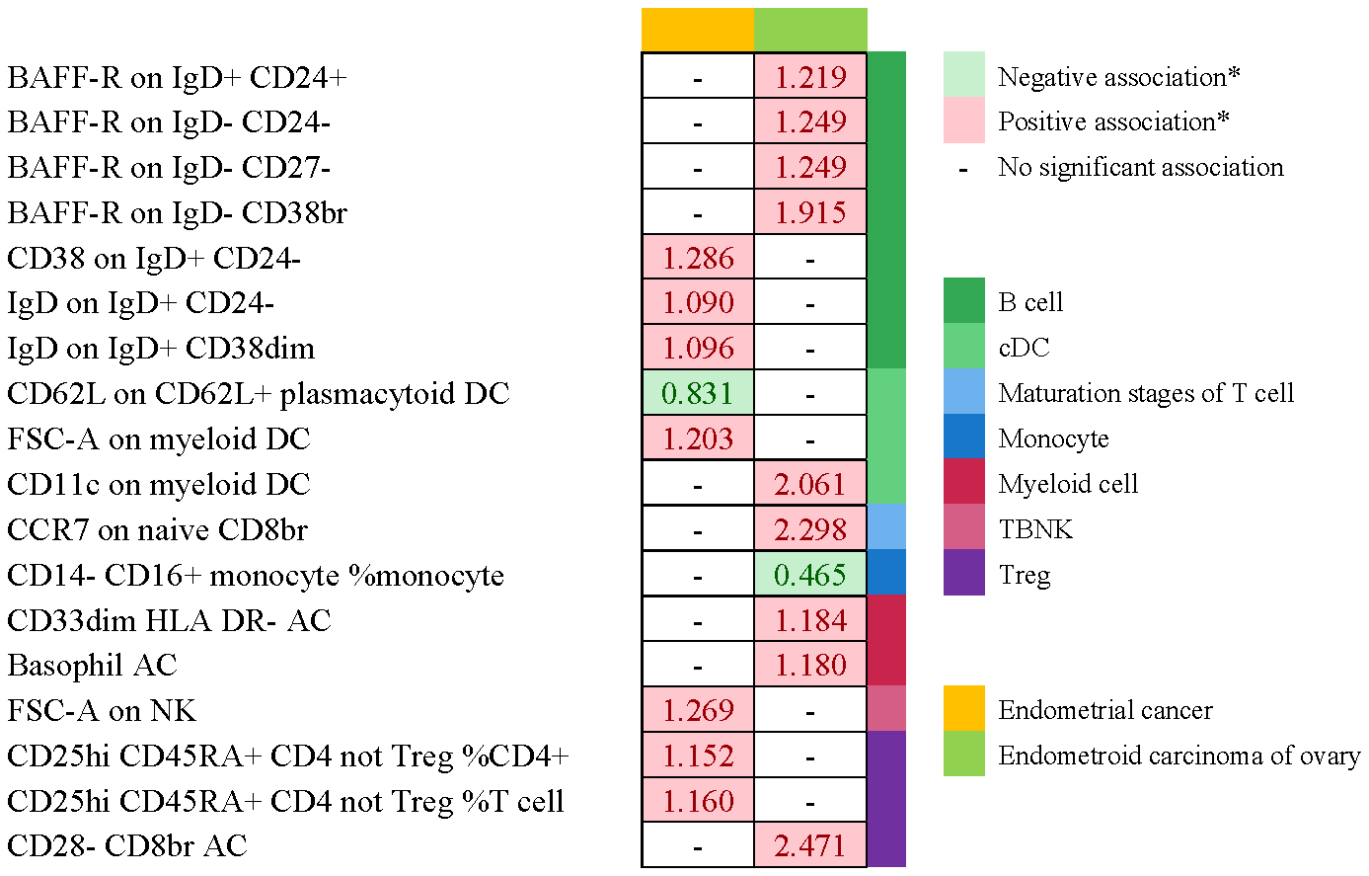
Summary of associations of genetically predicted immunophenotype traits with endometrial cancer. * means “P value <0.05, but 0.05< adj.P value <0.2 after FDR correction.

**Figure 10:**
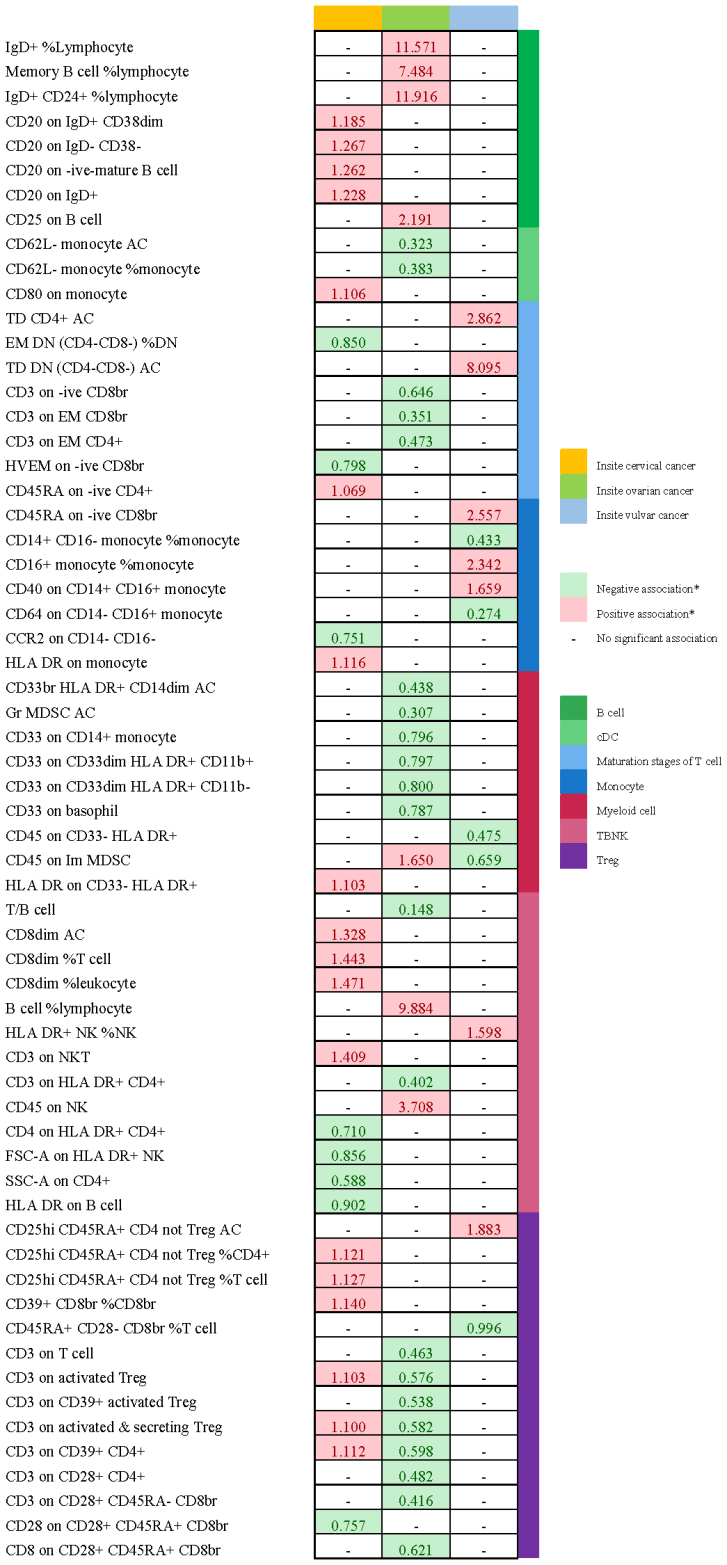
Summary of associations of genetically predicted immunophenotype traits with gynecologic carcinoma *in situ*. * means “P value <0.05, but 0.05< adj.P value <0.2 after FDR correction.

Endometrial carcinoma *in situ* was revealed to be causally related to 28 possible immunophenotypes (**Figure 7B**, **Supplementary Table 5**). The Treg panel’s characteristics were still prominent in endometrial cancer *in situ*, similar to carcinoma *in situ* of the cervix uteri.CD3 on T cell (OR=0.463, 95%CI:0.26-0.827, P=0.009), CD3 on activated CD4 regulatory T cell (OR=0.576, 95%CI:0.377-0.882, P=0.011), CD3 on CD39^+^ activated CD4 regulatory T cell (OR=0.538, 95%CI:0.347-0.835, P=0.006), CD3 on activated & secreting CD4 regulatory T cell (OR=0.582, 95%CI:0.382-0.884, P=0.011), CD3 on CD39^+^ CD4^+^ T cell (OR=0.598, 95%CI:0.394-0.909, P=0.016), CD3 on CD28^+^ CD4^+^ T cell (OR=0.482, 95%CI:0.276-0.842, P=0.01), CD3 on CD28^+^ CD45RA^-^ CD8^+^ T cell (OR=0.416, 95%CI:0.215-0.804, P=0.009), and CD8 on CD28^+^ CD45RA^+^ CD8^+^ T cell (OR=0.621, 95%CI:0.415-0.931, P=0.021) were inversely correlated with the incidence of endometrium carcinoma *in situ*. In Myeloid cell panel, CD45 on Immature Myeloid-Derived Suppressor Cells (OR=1.65, 95%CI:1.016-2.679, P=0.043) was linked in a higher incidence risk, while CD33^+^ HLA DR^+^ CD14dim, Granulocytic Myeloid-Derived Suppressor Cells, CD33 on CD14^+^ monocyte, CD33 on CD33^dim^ HLA DR^+^ CD11b^+^, CD33 on CD33dim HLA DR^+^ CD11b^-^, and CD33 on basophil were protective factors for carcinoma *in situ* of endometrium. We also found that there were causal links between B cells, T cell and cDC.

In carcinoma *in situ* of vulva, we identified 11 possible immunophenotypes (**Figure 7C**, **Supplementary Table 5**). HLA DR^++^ monocyte, CD20 on IgD^+^ CD24^+^ B cell, CD4 on HLA DR^+^ CD4^+^ T cell, HLA DR on CD14^+^ CD16^-^ monocyte, HLA DR on CD14^+^ monocyte, HLA DR on monocyte, and HLA DR on CD33^+^ HLA DR^+^ CD14^-^ had a negative association with vulvar carcinoma *in situ*. The following factors might raise the incidence of vulvar cancer *in situ*: CD25^++^ CD45RA^+^ CD4 not regulatory T cell, Terminally Differentiated CD4^+^ T cell, CD16^+^ monocyte, HLA DR^+^ Natural Killer cell, CD40 on CD14^+^ CD16^+^ monocyte, and CD45RA on naive CD8^+^ T cell.

### 3.7 Sensitivity analysis

Sensitivity analysis was performed to ensure the robustness of the causal evaluation because IVW methods are prone to weak instrumental bias. The highly probable or possible immunophenotypes showed no evidence of horizontal pleiotropy when examined using the MR-Egger intercept method (all P > 0.05). Cochran’s Q statistic test did not reveal any evidence of heterogeneity (all P > 0.05). The MR Steiger directionality test additionally demonstrated the causative links between immune cells and gynecologic malignancies. In our results, reverse causality was not observed. (**Supplementary Tables 7**).

## 4 Discussion

**Figure 11:**
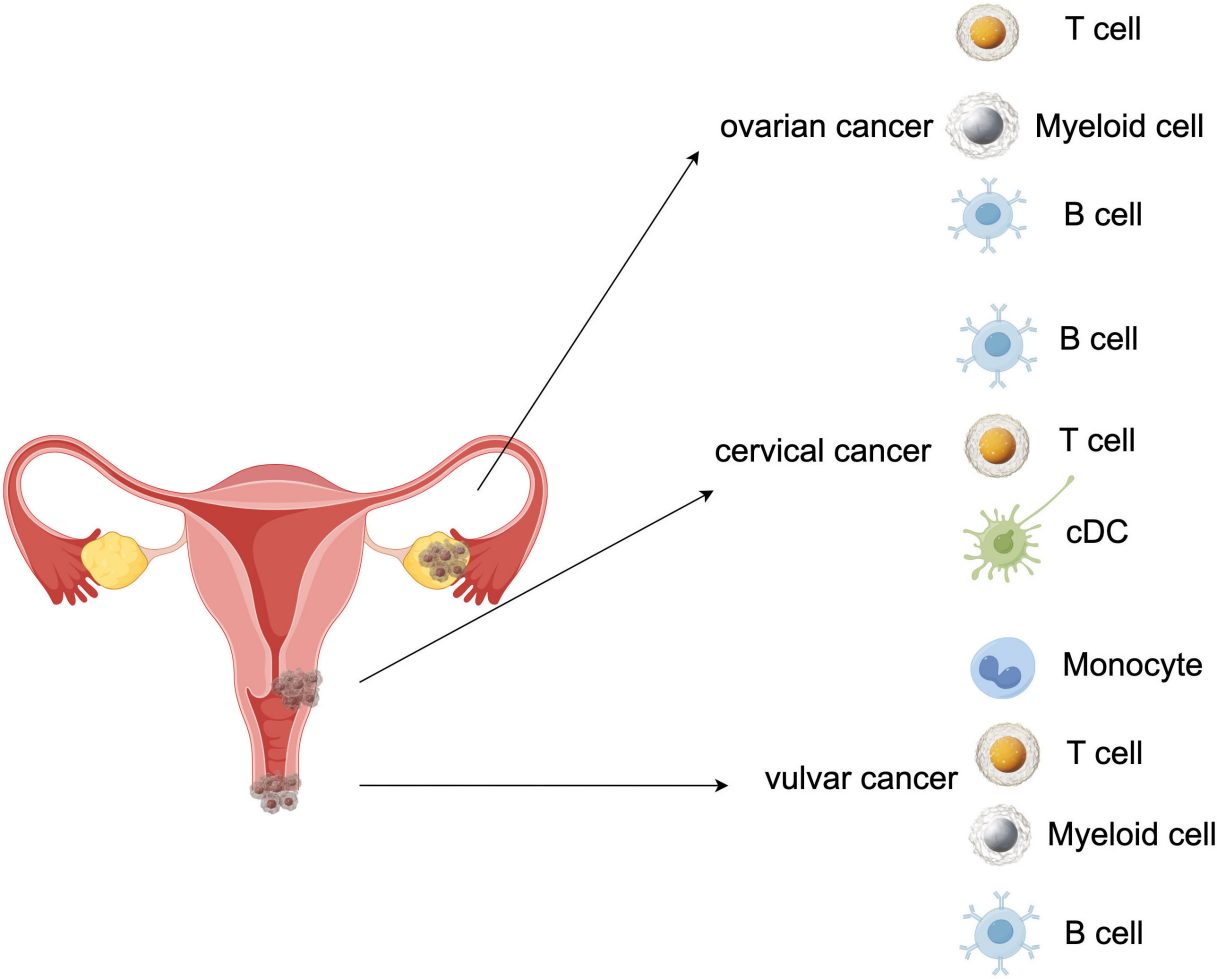
A schematic summary figure for the positive results after the Bonferroni method. (Created with Figdraw). **(A)** Three important assumptions for choosing instrumental variables (IVs); **(B)** The overview of the study design.

The three most prevalent types of gynecological malignancies are cervical cancer, endometrial cancer, and ovarian cancer (1). Besides endangering a woman’s ability to conceive, the gynecological cancers can be fatal in their advanced stages. In addition, female patients will suffer serious psychological harm as a result of the lesion’s location. More research is showing that immunological imbalance is necessary for cancer developing, but the function of the immune system in the progression of gynecological cancers is still unknown. In this study, we used a two-sample MR analysis to acquire information about the genetic evidence links between immunophenotypes and four gynecologic malignancies. With IVW techniques, we successfully managed to determine 65 possible immunophenotype traits and 50 highly probable ones. 19 traits in B cell panel, 8 in TBNK, 6 in cDC, monocyte, and maturation stages of T cell, 4 in myeloid cell, and 2 in Treg composed the highly probable immunophenotypes. In order to help the general public understand the excellent outcomes, we also plotted a schematic summary figure in **Figure 11**. The study revealed a more thorough and trustworthy causation of immunophenotype in gynecologic cancers than we anticipated.

We used 4 distinct MR analysis methods to conduct a large-scale MR analysis for this work. Initially, we searched into the potential connection between immune cells and four gynecologic cancers, including preinvasive carcinoma. 206 pairs of significant (P<0.05) causal associations were confirmed by the results of the MR analysis (**Supplementary Table 2**). Nevertheless, we modified the P-value to adj.P-value (FDR adjusted with Benjamini-Hochberg method) in regard to multiple comparisons. Our research revealed that 32 pairs had highly probable causative effects for cervical cancer, 9 for ovarian cancer, and 9 for vulvar cancer. Unfortunately, the findings failed to confirm a high probability of a causal link between immunophenotype traits and endometrial cancer. Thus, we identified the immunophenotypes that showed P value <0.05; and 0.05< adj. P value <0.2 was considered to be possible factors. Subsequently, 18 possible immunophenotypes for endometrial cancer and 65 for *in situ* gynecologic carcinoma were identified.

In assessing immunological components in the TME, T cells and myeloid cells have been the subject of numerous investigations. But little research has been done on the function of B cells. In our study, the B-cell panel showed the highest number of significant associations in cervical cancer when compared to other panels, and the majority of B cells panel (e.g. BAFF-R on CD24^+^ CD27^+^ B cell, BAFF-R on IgD^+^ CD24^+^ B cell, BAFF-R on IgD^+^ CD24- B cell,BAFF-R on IgD^+^ CD38- B cell, CD19 on CD24^+^ CD27^+^ B cell, and CD19 on memory B cell) were protective factors against cervical cancer. A recent investigation provided evidence that B-cells performed an anti-tumorigenic effect on squamous cell carcinomas associated with HPV. Furthermore, the findings demonstrated that B-cell specific molecule (CD19) was a predictive survival biomarker in head and neck squamous cell carcinoma and cervical squamous cell carcinomas (32). Additionally, Kim SS et al. discovered that B-cell depleted mice had larger tumors and grew at a faster rate than matched mice with controls, indicating a critical function for B-cells in the progression of squamous cell carcinoma (32). Cao and colleagues mapped the immunological landscape of cervical cancer using single-cell RNA sequencing. The findings demonstrated that germinal center B cells improved clinical outcomes and have anti-tumor abilities (33). The diversity of B-cell subsets in anti-tumor responses was also demonstrated by Cao et al. Our findings were consistent with the aforementioned studies, which show that B cells significantly improved the prognosis of cervical cancer patients.

Prior studies have indicated that the lymphocyte-to-monocyte ratio (LMR) has been explored as a potential predictive marker for ovarian cancer. Similarly, we discovered that monocytes (such as HLA DR on CD14^+^ CD16-monocyte, HLA DR on CD14^+^ monocyte, and HLA DR on monocyte) were linked to a lower risk of ovarian cancer. According to a clinical trial, patients with a high LMR typically respond better to chemotherapy, and the complete response (CR) rate differed significantly between the LMR-low and LMR-high groups. (48.9% vs. 75.3%, P < 0.0001) (34). In patients with ovarian cancer, low LMR was linked with poor survival outcomes, particularly poor OS and PFS, based on the findings of a meta-analysis (35). Consistent with previous research, our results showed that monocytes were positively correlated with survival and may contribute to maintaining the equilibrium between anti-tumor immune response and tumor promoting capacity.

This study is the first to investigate the causative relationships between immunophenotype traits and gynecologic malignancies via a two-sample Mendelian randomization. The study’s main strength is the MR method, which eliminated bias from other variables and reverse causality. Our analysis’s broad coverage of immune cells and large sample size, which outperformed comparable observational studies in terms of statistical efficiency, are two of its main advantages. An additional benefit is that our study was limited to European participants, thereby decreasing the possibility of heterogeneity. Thirdly, all IVs satisfied the criterion that F-statistics > 10, confirming no weak IVs bias. Yet it is also essential to note our study’s limitations. First off, since all the GWAS summary data were from European populations, more research is needed to determine whether our findings apply to other racial or ethnic groups. Second, we are unable to do a stratified analysis of the population in the absence of baseline information (such as age, gender, TNM stage, and grade), which could potentially muddy the causal link due to hidden population structure. Thirdly, there are fewer SNPs accessible for some immunophenotype features in this because of the stringent screening IV cut-off, which could have resulted in bias.

## 5 Conclusions

To sum up, we discovered that the level of various immunophenotypes was connected to a risk of gynecologic malignancies based on a bidirectional two-sample MR study. Our study presented intriguing results on the causative relationship between immunological factors and gynecologic cancers. According to our findings, immune cell-targeting lymphocyte subset harmonies may be a viable intervention strategy for the prevention of gynecologic cancers. These findings also offer compelling justification for the creation of new immune cell-targeting therapies and additional methods for diagnosis.

## Data availability statement

The datasets presented in this study can be found in online repositories. The names of the repository/repositories and accession number(s) can be found in the article/**Supplementary Material**.

## Ethics statement

All studies included in cited genome-wide association studies had approved by a relevant review board. All participants had provided the inform consent. FinnGen research project is based on samples from Finnish biobanks and data from national health registers and applies the permissions to utilize the register data for research purposes from national authorities. All studies were conducted in accordance with the Declaration of Helsinki.

## Author contributions

YL: Conceptualization, Formal analysis, Funding acquisition, Supervision, Writing – review & editing. JL: Formal analysis, Methodology, Software, Visualization, Writing – original draft. QW: Data curation, Software, Writing – review & editing. YZ: Visualization, Writing – original draft. CZ: Data curation, Writing – review & editing. JP: Funding acquisition, Methodology, Supervision, Validation, Writing – review & editing.
