# Supplementary material for: The causal effects of genetically determined immune cells on gynecologic malignancies: a Mendelian randomization study
Source: Front Oncol. 2024 Apr 30;14:1371309. doi: 10.3389/fonc.2024.1371309 (PMC11091348; doi:10.3389/fonc.2024.1371309)
Supplement: Supplementary file 1 [file DataSheet_1.zip › Supplementary Material/Supplementary_Figure.docx]

Supplementary Material

## Supplementary Figures


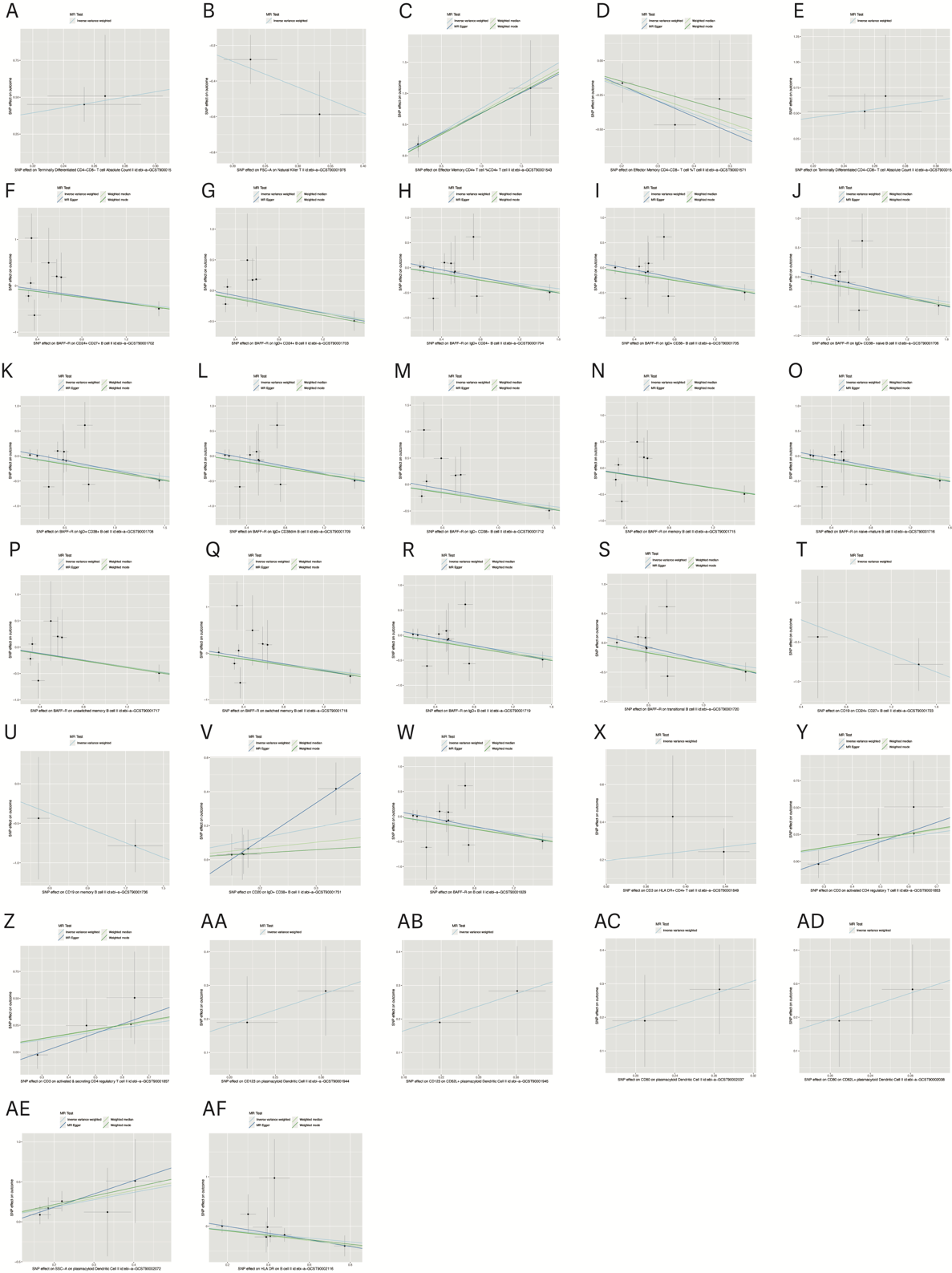


Supplementary Figure1. Scatter plot for the relationship between the SNP effect size of causal immune traits (x-axis) and the corresponding effect size estimates of cervical cancer (y-axis).


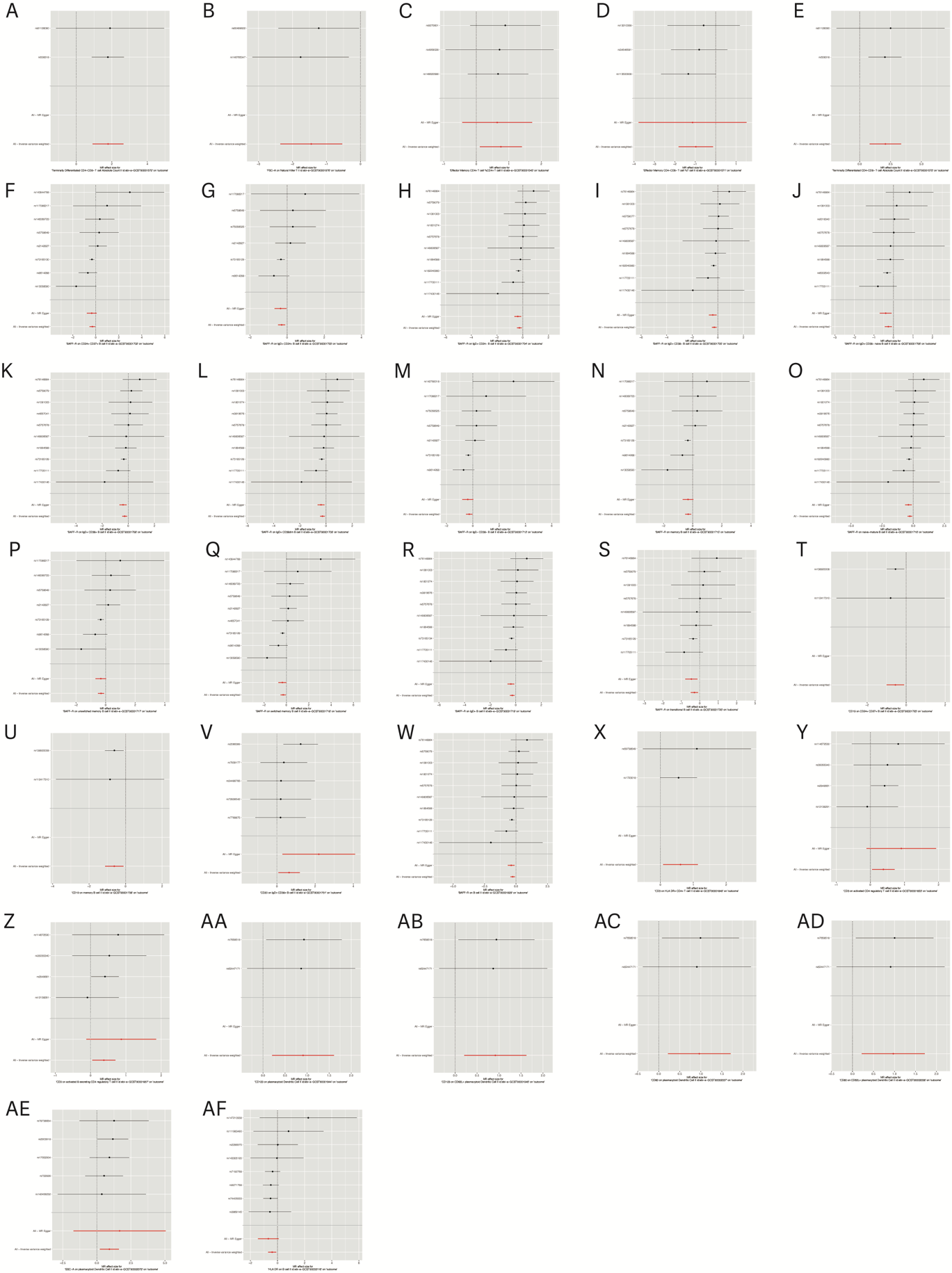


Supplementary Figure2. Forest plot for the relationship between the immune traits and cervical cancer by MR analysis. The significance of red lines are MR results of inverse variance weighted method and/or MR Egger test.


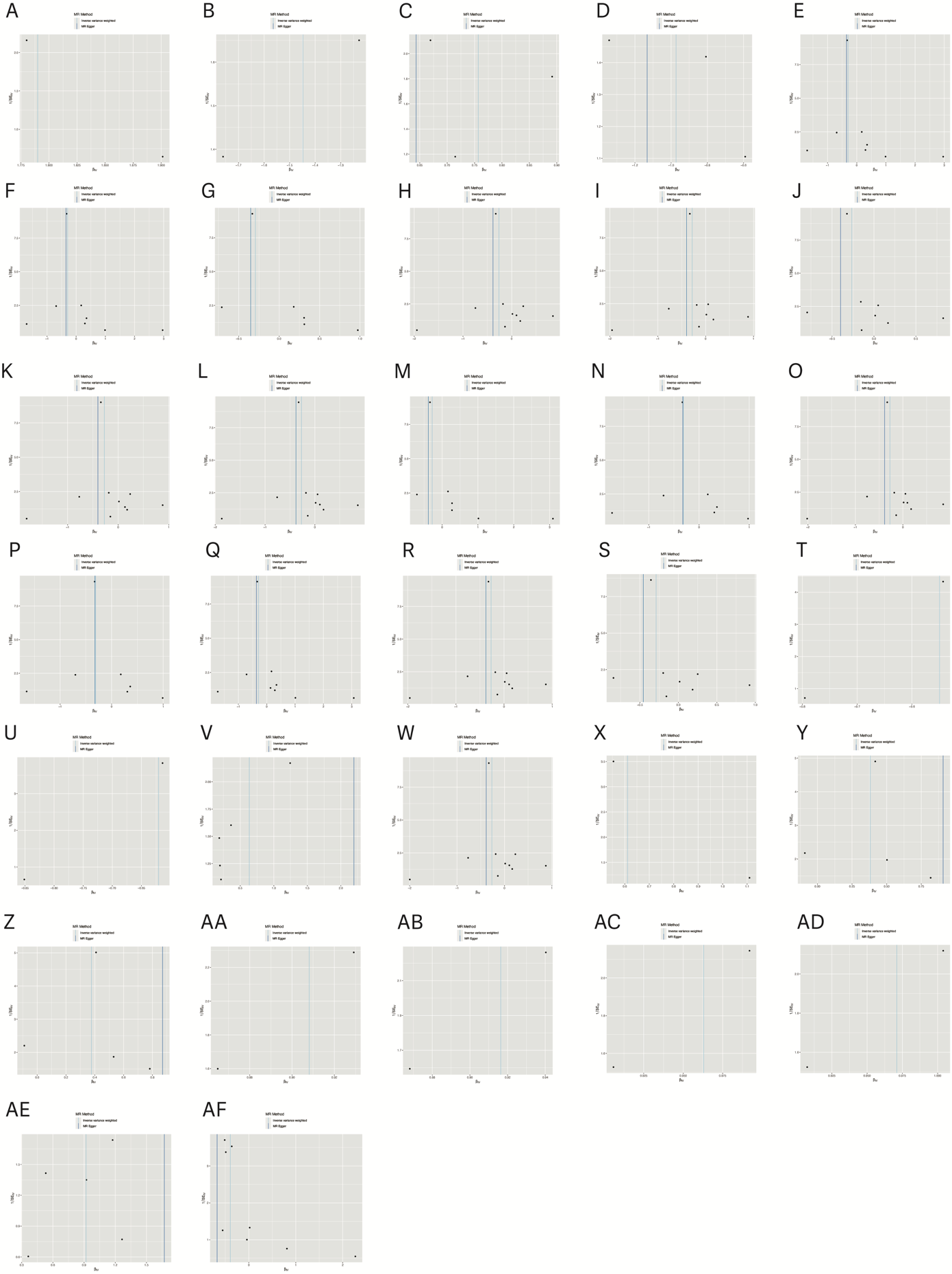


Supplementary Figure3. Funnel plot of immune traits on cervical cancer. The funnel plots are symmetric, which shows that the absence of heterogeneity.


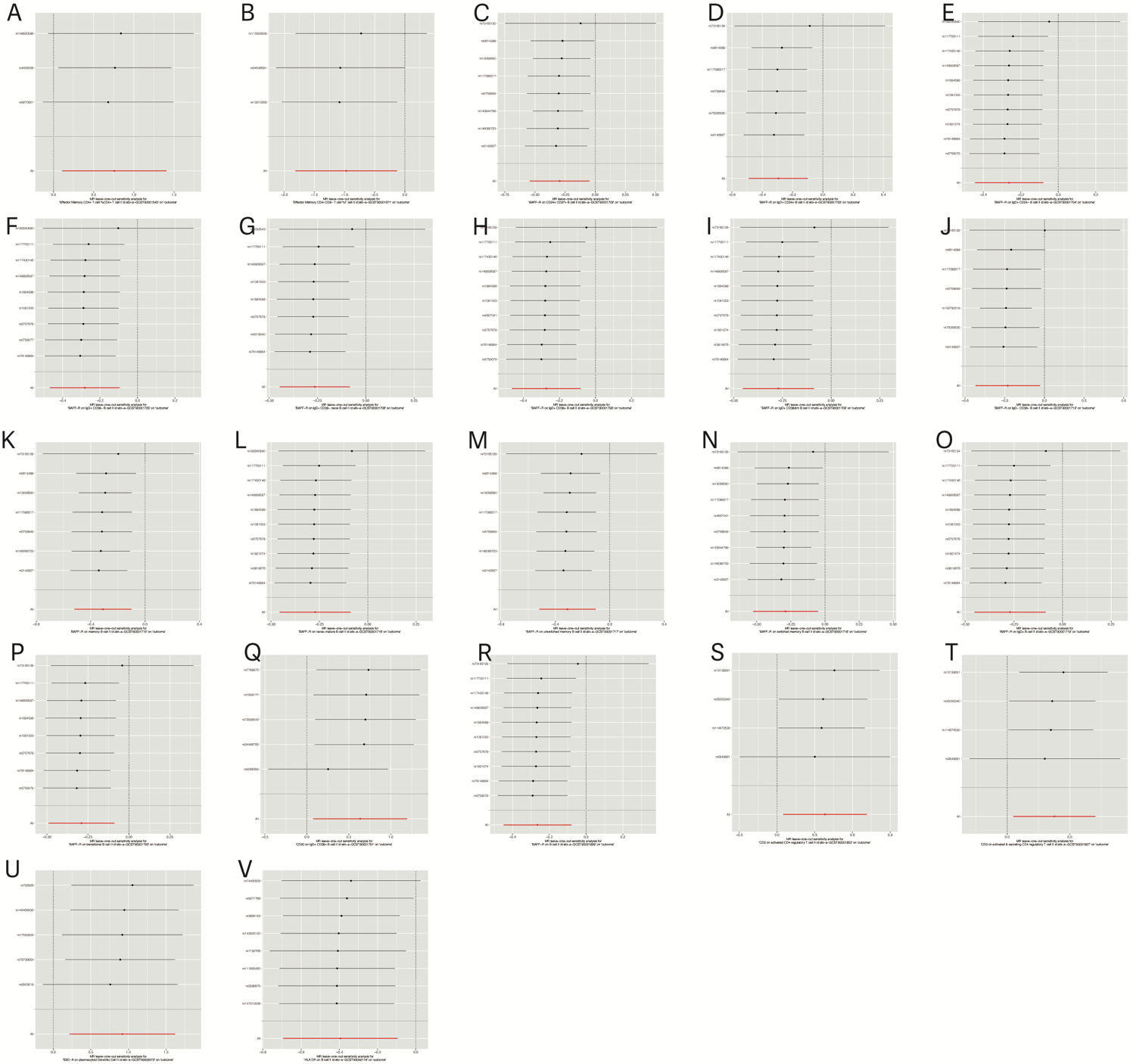


Supplementary Figure4 Leave-one-out analysis result of SNPs associated with dried fruit intake and risk on cervical cancer.


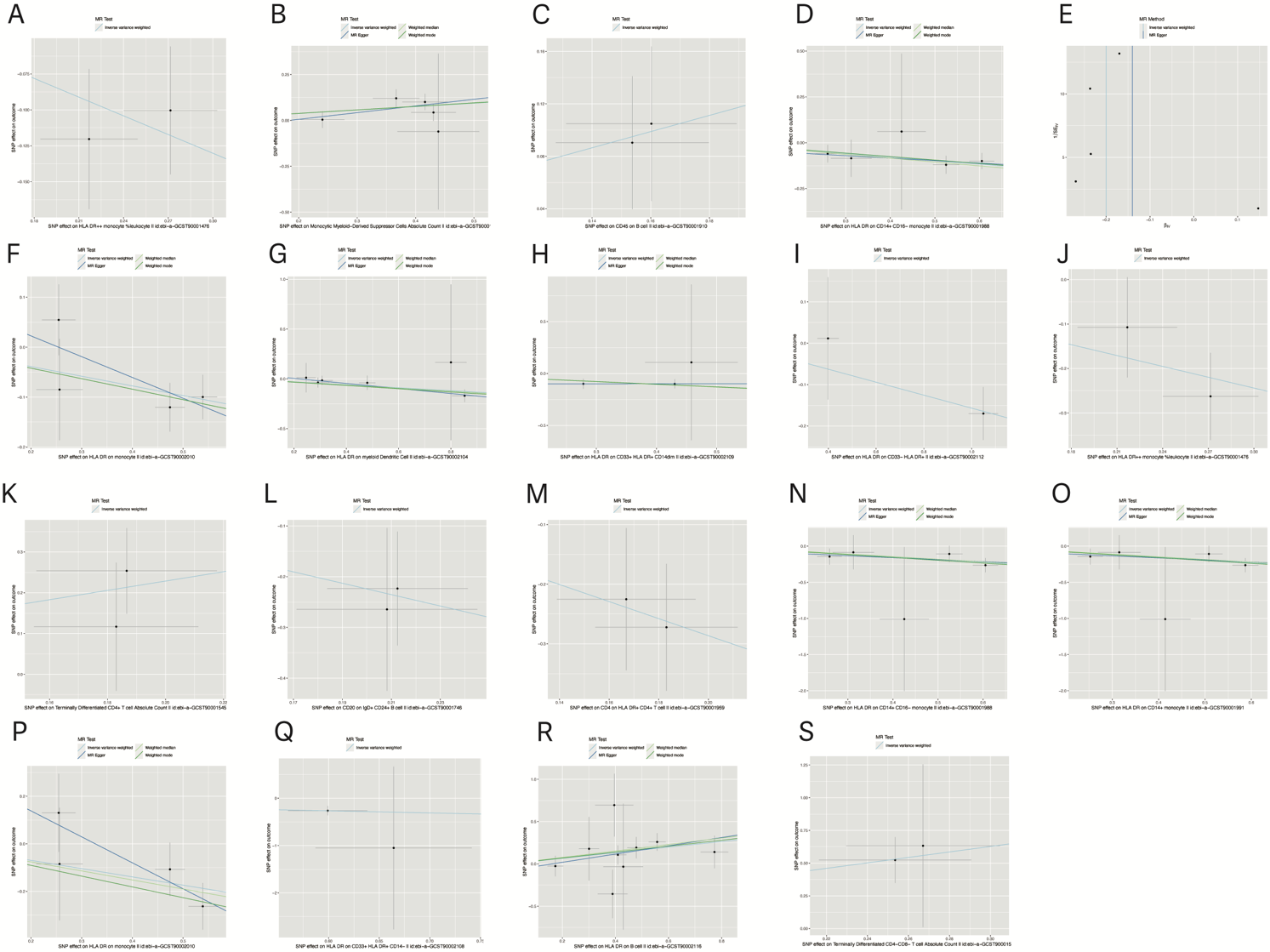


Supplementary Figure5. Scatter plot for the relationship between the SNP effect size of causal immune traits and the corresponding effect size estimates of ovarian cancer(A-I), vulvar cancer (J-R) and Carcinoma in situ of vulva(S).


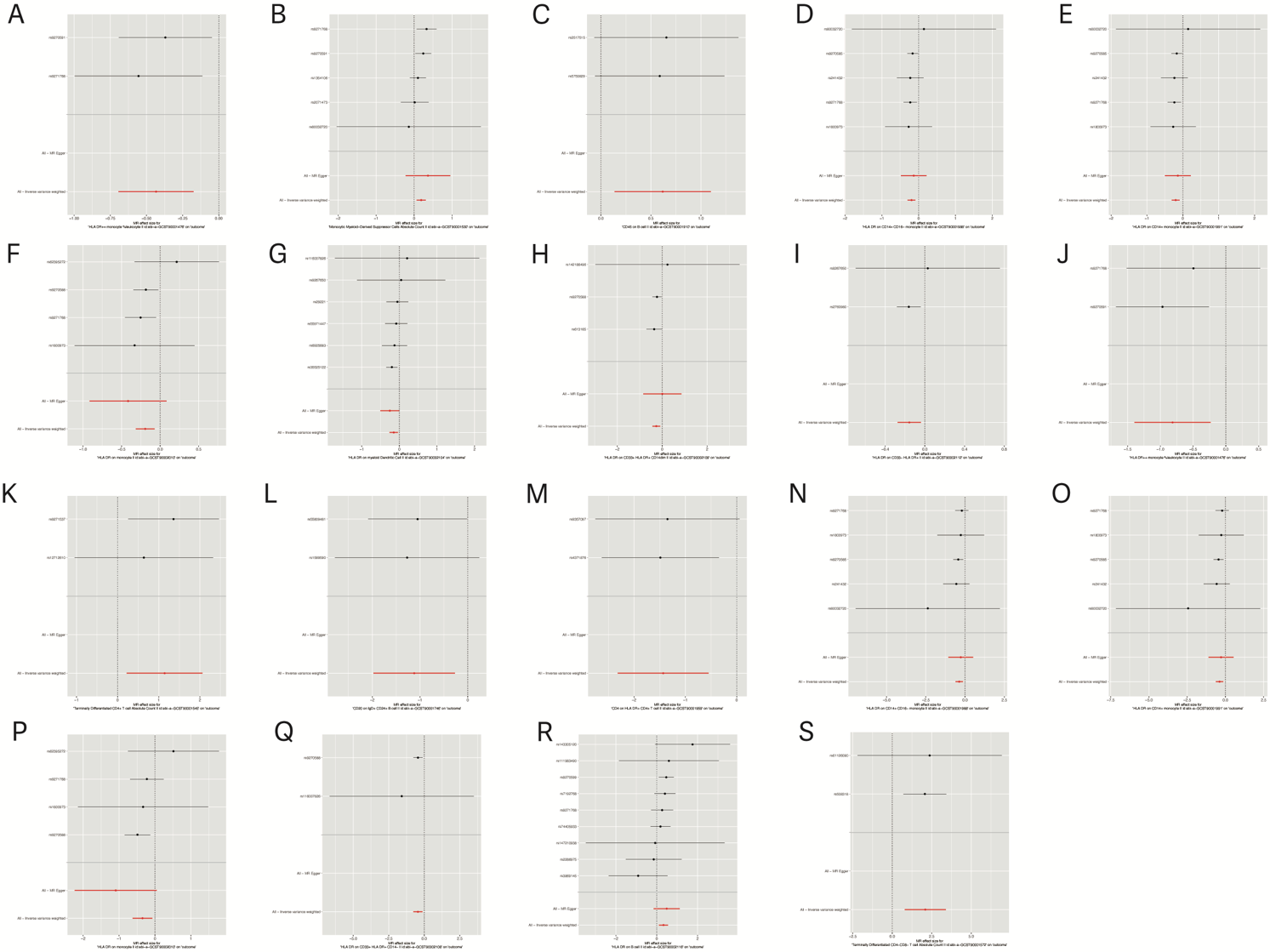


Supplementary Figure6. Forest plot for the relationship between the immune traits and ovarian cancer (A-I), vulvar cancer (J-R) and Carcinoma in situ of vulva(S) by MR analysis. The significance of red lines are MR results of inverse variance weighted method and/or MR Egger test.


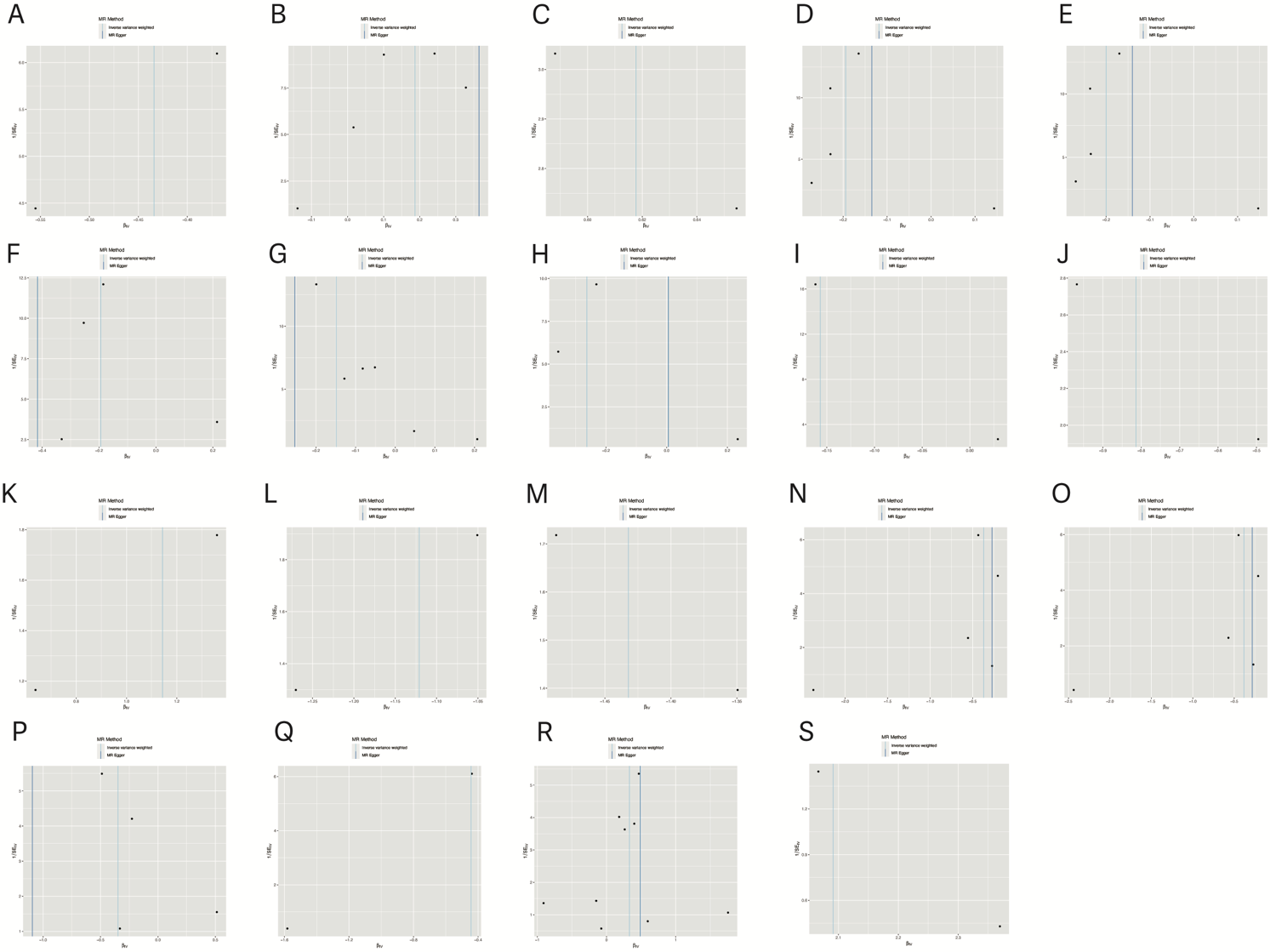


Supplementary Figure7. Funnel plot of immune traits on ovarian cancer(A-I), vulvar cancer(J-R) and Carcinoma in situ of vulva(S). The funnel plots are symmetric, which shows that the absence of heterogeneity.


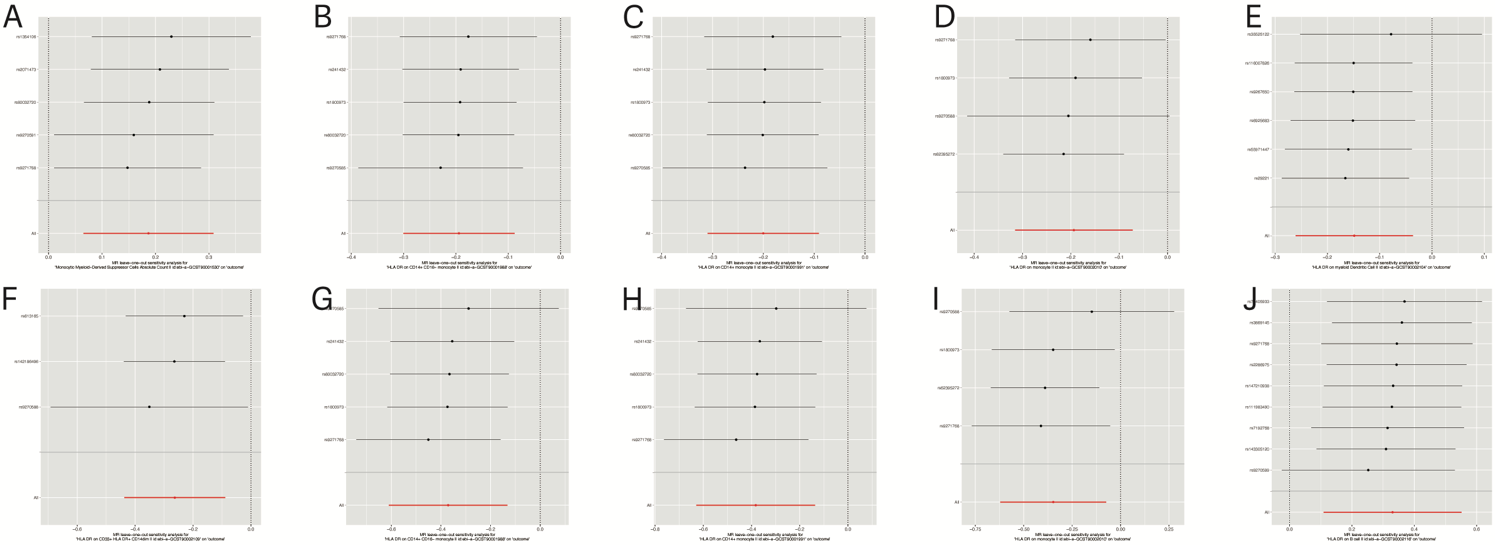


Supplementary Figure8. Leave-one-out analysis result of SNPs associated with dried fruit intake and risk on ovarian cancer(A-I), vulvar cancer(J-R) and Carcinoma in situ of vulva(S).
