# Supplementary figures and images for: The causal effects of genetically determined immune cells on gynecologic malignancies: a Mendelian randomization study
Source: Front Oncol. 2024 Apr 30;14:1371309. doi: 10.3389/fonc.2024.1371309 (PMC11091348; doi:10.3389/fonc.2024.1371309)

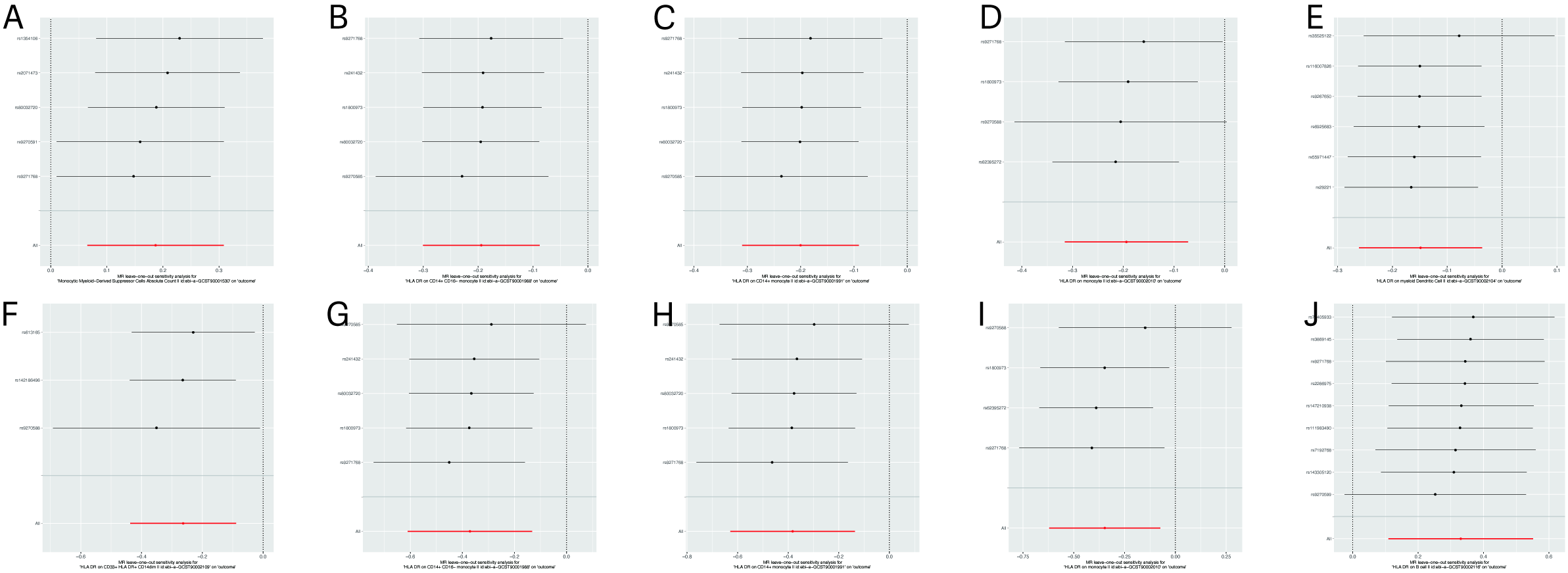

Supplement: Supplementary file 1 [file DataSheet_1.zip › Supplementary Material/FS8.tif]

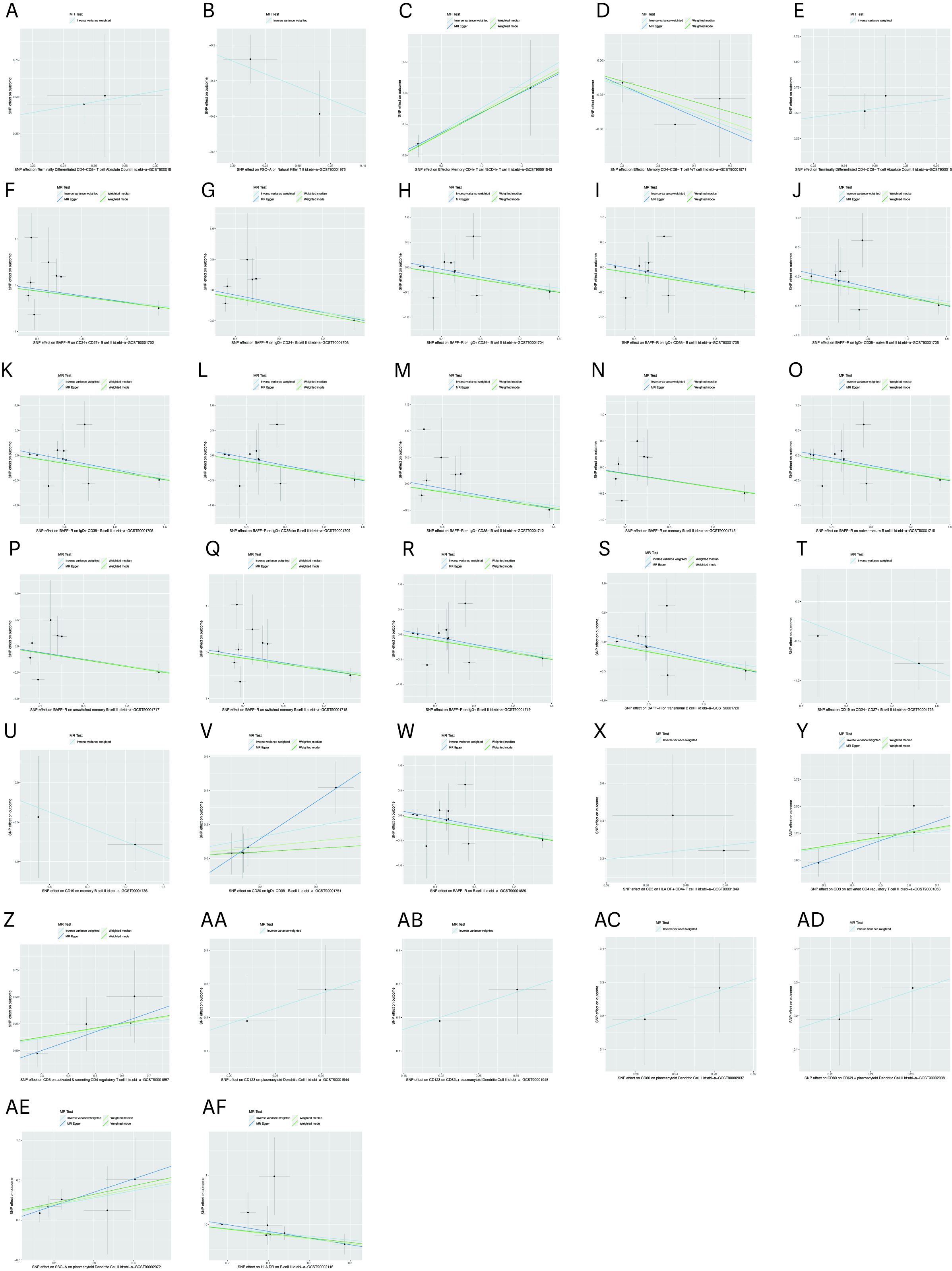

Supplement: Supplementary file 1 [file DataSheet_1.zip › Supplementary Material/FS1.tif]

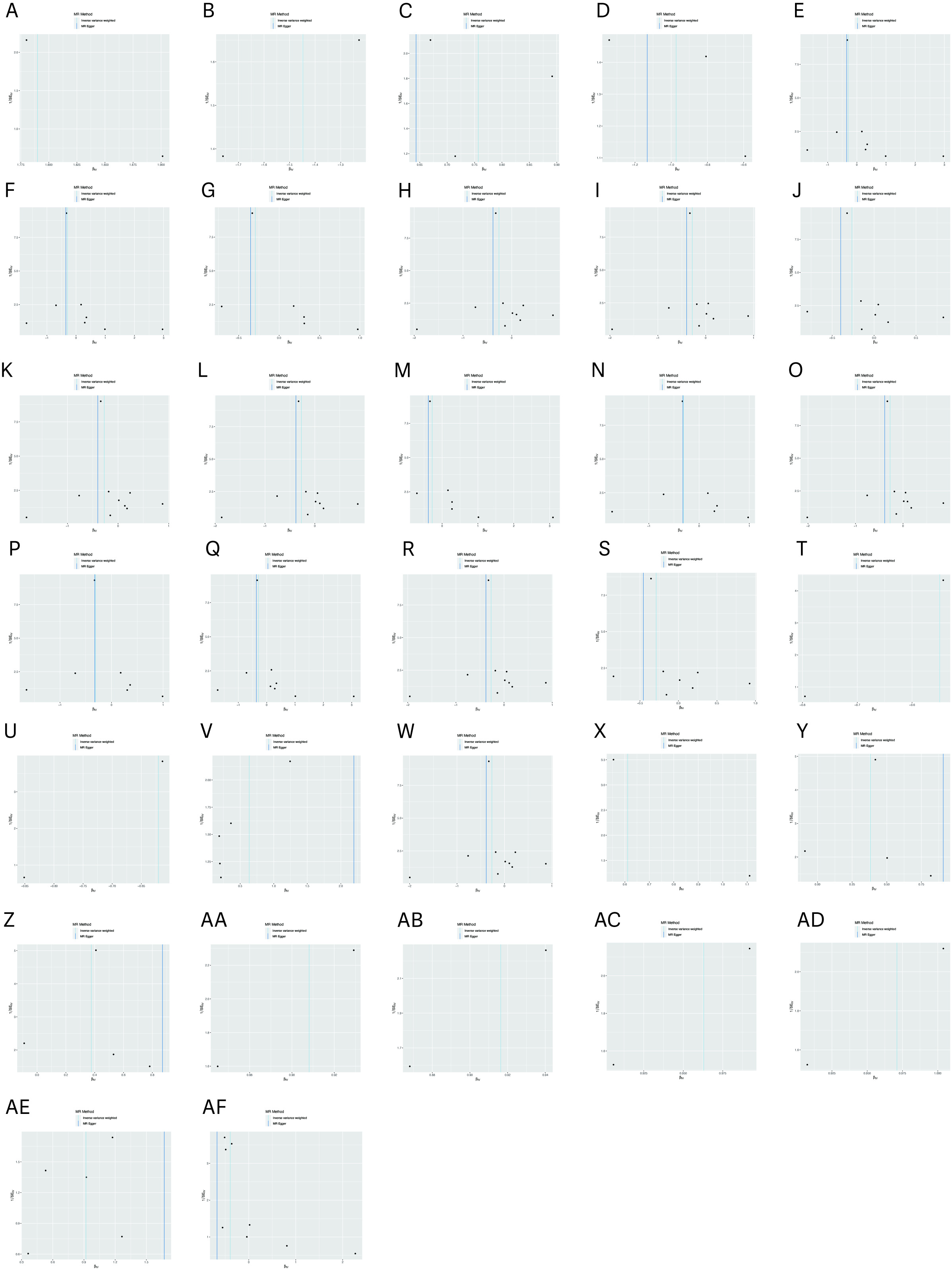

Supplement: Supplementary file 1 [file DataSheet_1.zip › Supplementary Material/FS3.tif]

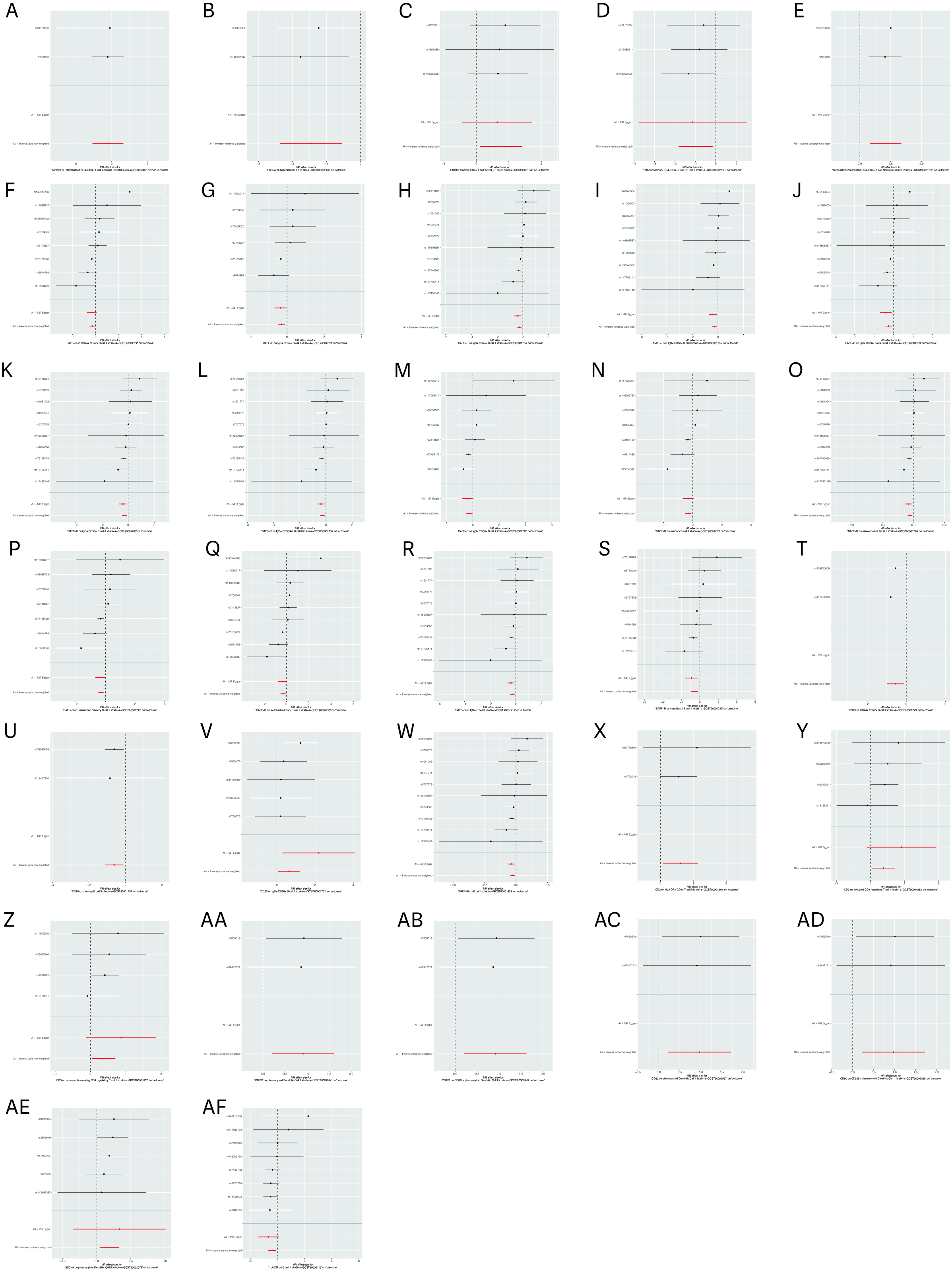

Supplement: Supplementary file 1 [file DataSheet_1.zip › Supplementary Material/FS2.tif]

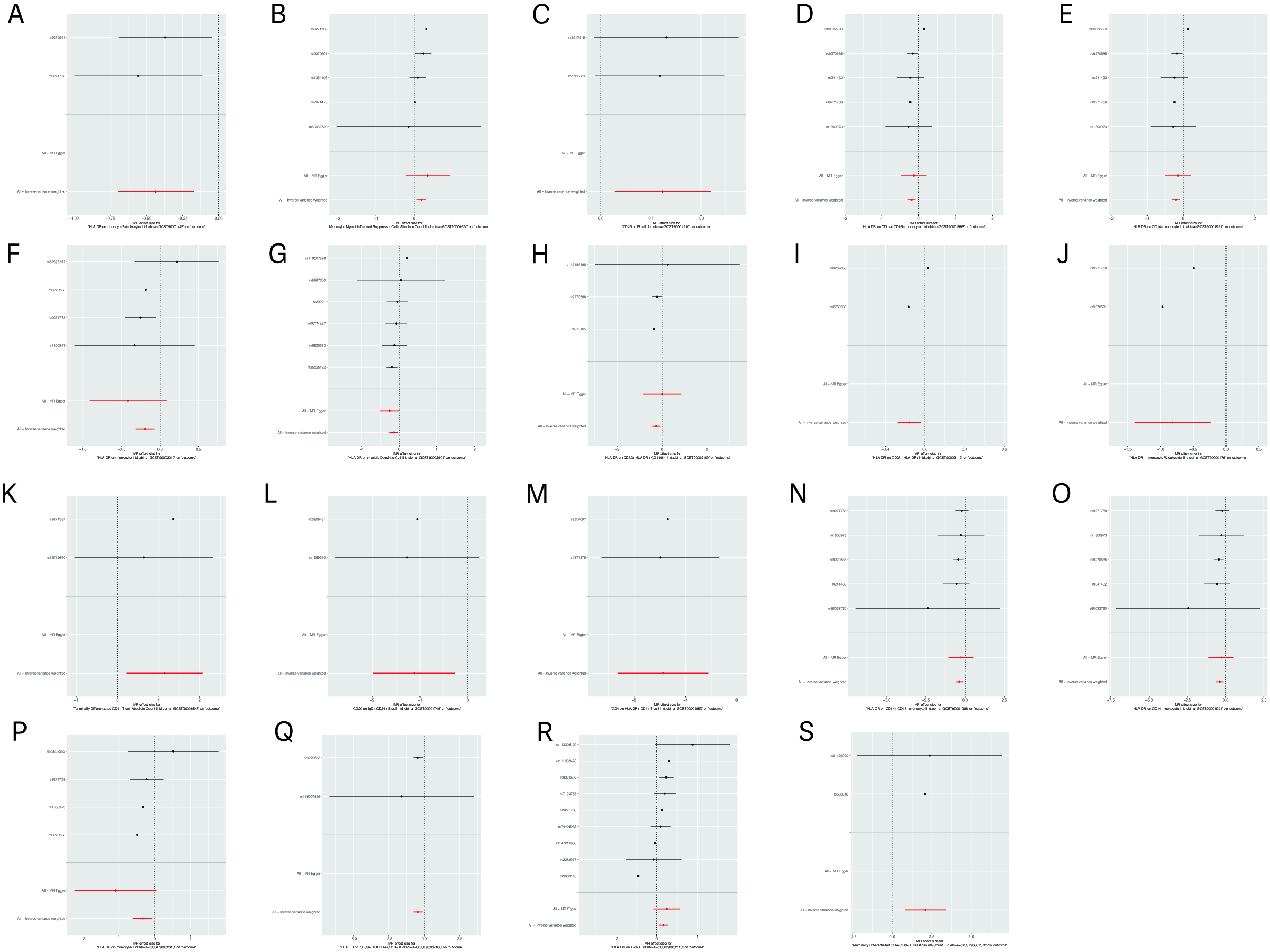

Supplement: Supplementary file 1 [file DataSheet_1.zip › Supplementary Material/FS6.tif]

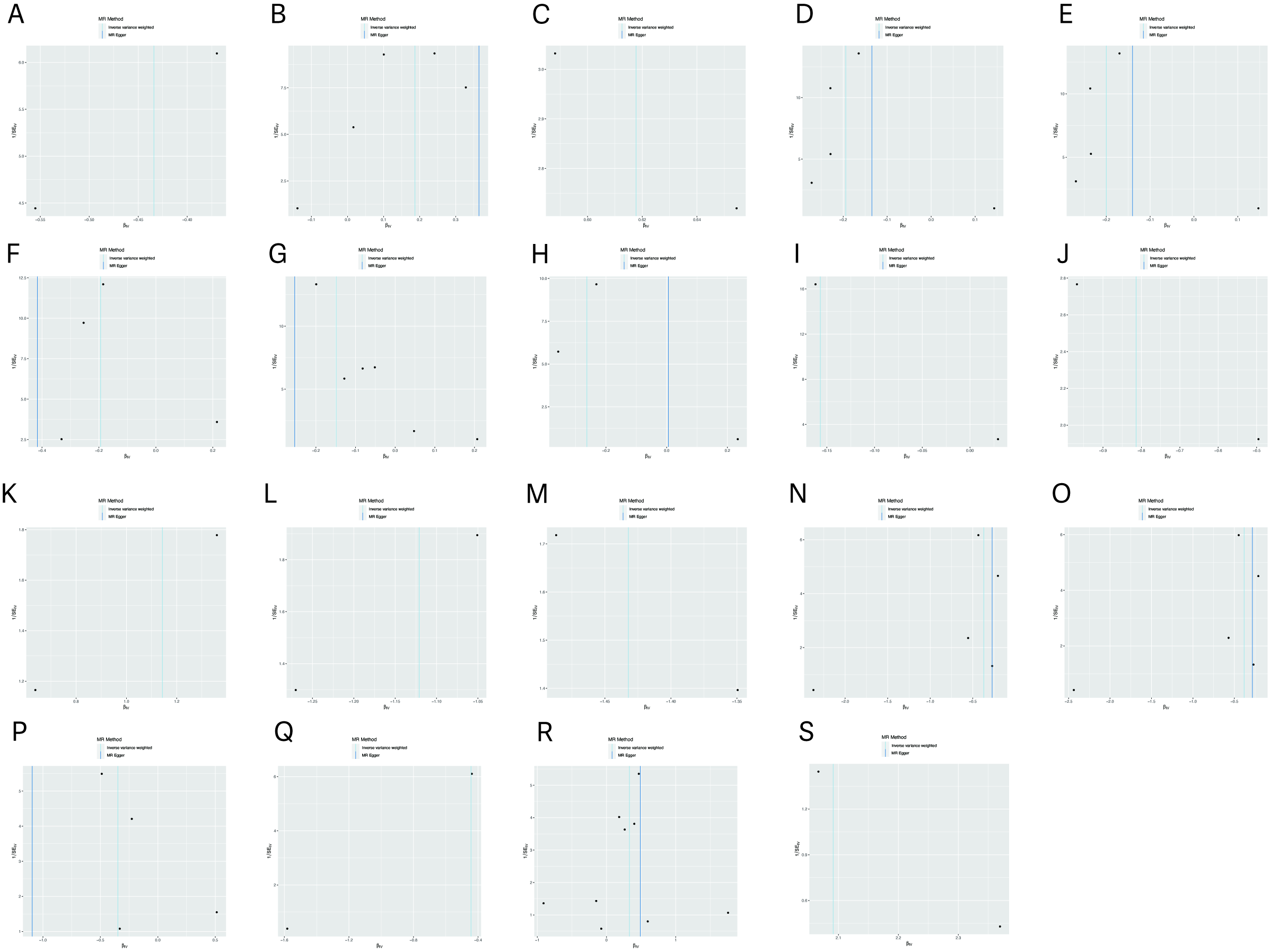

Supplement: Supplementary file 1 [file DataSheet_1.zip › Supplementary Material/FS7.tif]

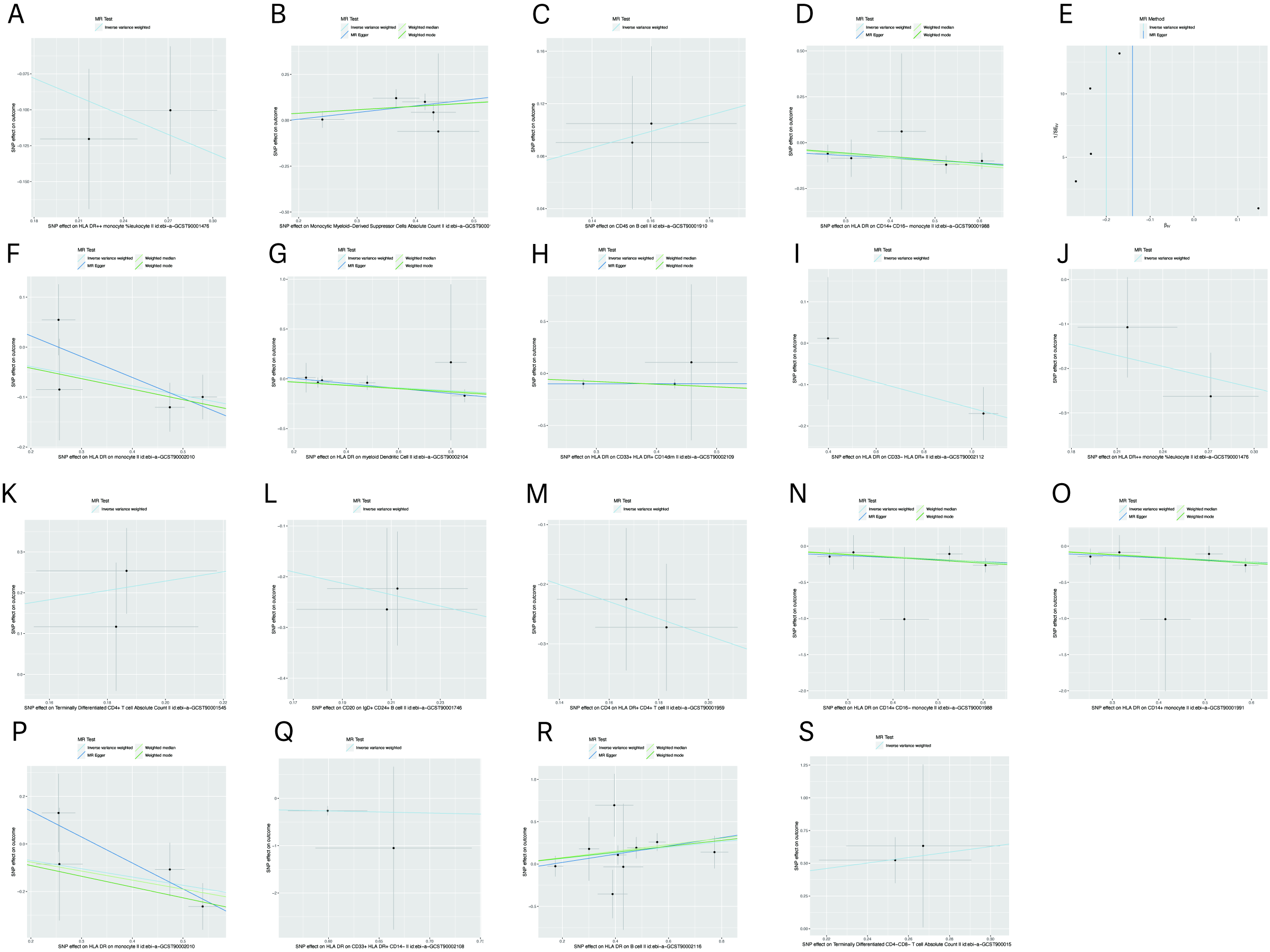

Supplement: Supplementary file 1 [file DataSheet_1.zip › Supplementary Material/FS5.tif]

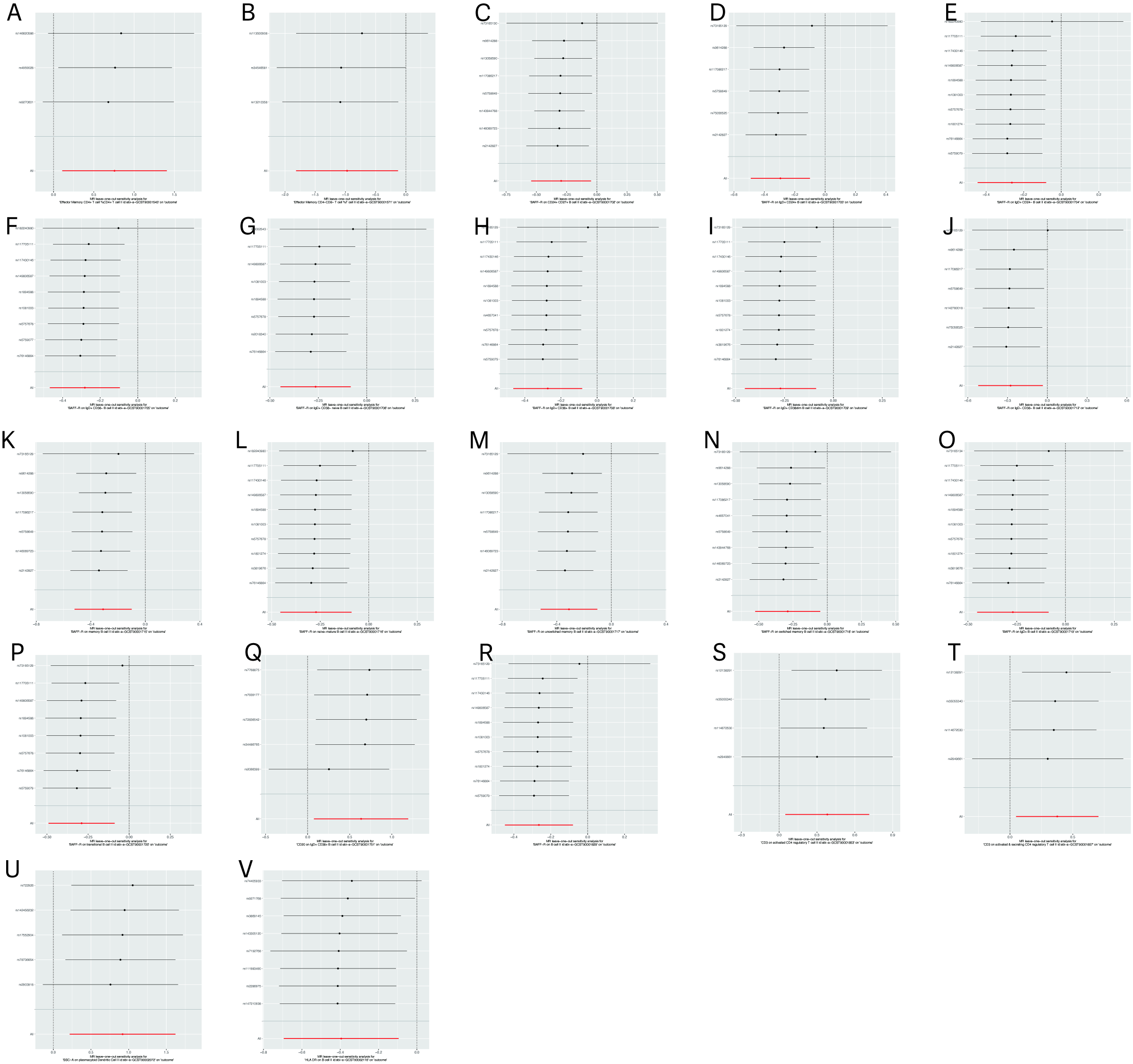

Supplement: Supplementary file 1 [file DataSheet_1.zip › Supplementary Material/FS4.tif]
